# Supplementary material for: Associations between fruit and vegetable consumption and HCC occurrence in patients with cirrhosis
Source: JHEP Rep. 2025 Feb 13;7(5):101355. doi: 10.1016/j.jhepr.2025.101355 (PMC12008579; doi:10.1016/j.jhepr.2025.101355)
Supplement: Multimedia component 1 [file mmc1.pdf]

Associations between fruit and vegetable consumption and incident hepatocellular carcinoma among patients with cirrhosis

Florian Manneville, Zineb Zouakia, Séverine Donneger, Leopold K. Fezeu, Alice Bellocchi, Pierre Nahon, Mathilde Touvier, Nathalie Ganne-Carrié, Chantal Julia

Table of contents

Fig. S1. 2

Fig. S2. 4

Fig. S3. 6

Table S1. 8

Table S2. 9

Table S3. 10

Table S4 11

Table S5. 13

Table S6. 14

Table S7. 15

Table S8 16

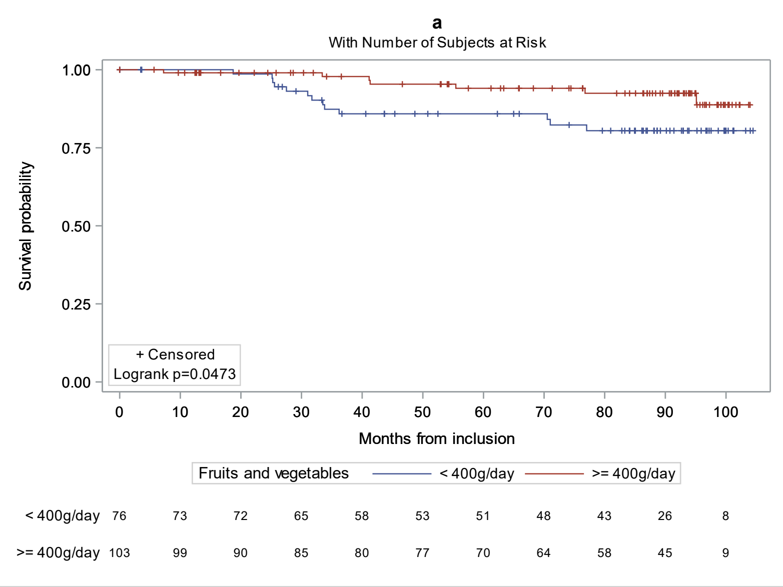
a
With Number of Subjects at Risk
Survival probability
1.00
0.75
0.50
0.25
0.00
+ Censored
Logrank p=0.0473
0 10 20 30 40 50 60 70 80 90 100
Months from inclusion
Fruits and vegetables
< 400g/day
>= 400g/day
< 400g/day 76 73 72 65 58 53 51 48 43 26 8
>= 400g/day 103 99 90 85 80 77 70 64 58 45 9

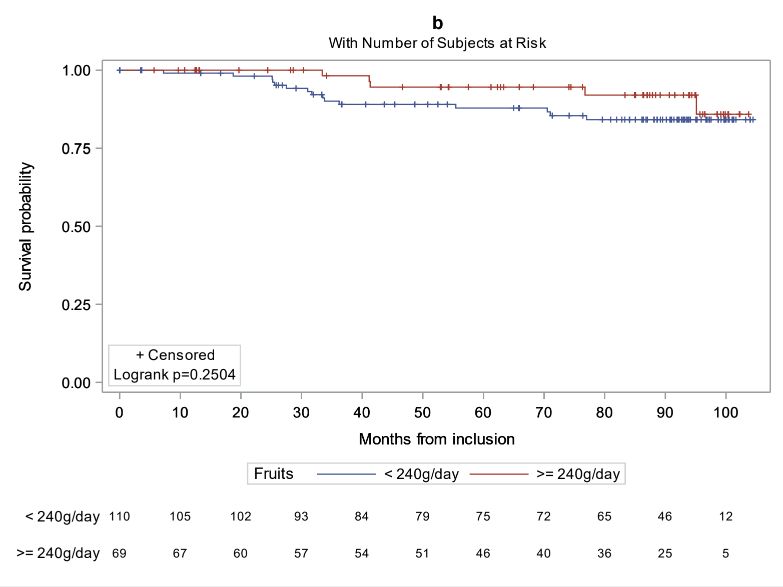
b
With Number of Subjects at Risk
Survival probability
1.00
0.75
0.50
0.25
0.00
+ Censored
Logrank p=0.2504
0 10 20 30 40 50 60 70 80 90 100
Months from inclusion
Fruits
< 240g/day
>= 240g/day
< 240g/day 110 105 102 93 84 79 75 72 65 46 12
>= 240g/day 69 67 60 57 54 51 46 40 36 25 5

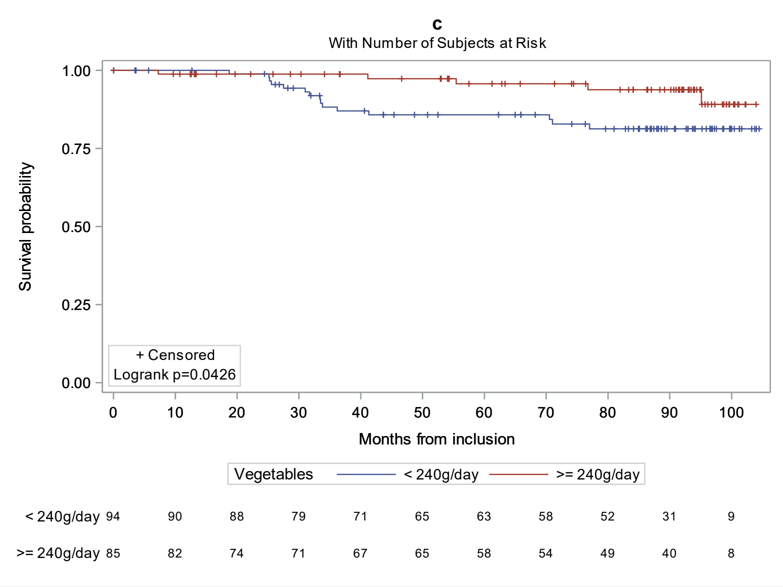
c
With Number of Subjects at Risk
Survival probability
1.00
0.75
0.50
0.25
0.00
+ Censored
Logrank p=0.0426
0 10 20 30 40 50 60 70 80 90 100
Months from inclusion
Vegetables
< 240g/day
>= 240g/day
< 240g/day 94 90 88 79 71 65 63 58 52 31 9
>= 240g/day 85 82 74 71 67 65 58 54 49 40 8

Fig. S1. Kaplan-Meier curves for incident HCC among patients with liver cirrhosis who consumed a) fruit and/or vegetable, b) fruit, and c) vegetable at or above thresholds (n=179). Level of significance: p= 0.05 (logrank test).

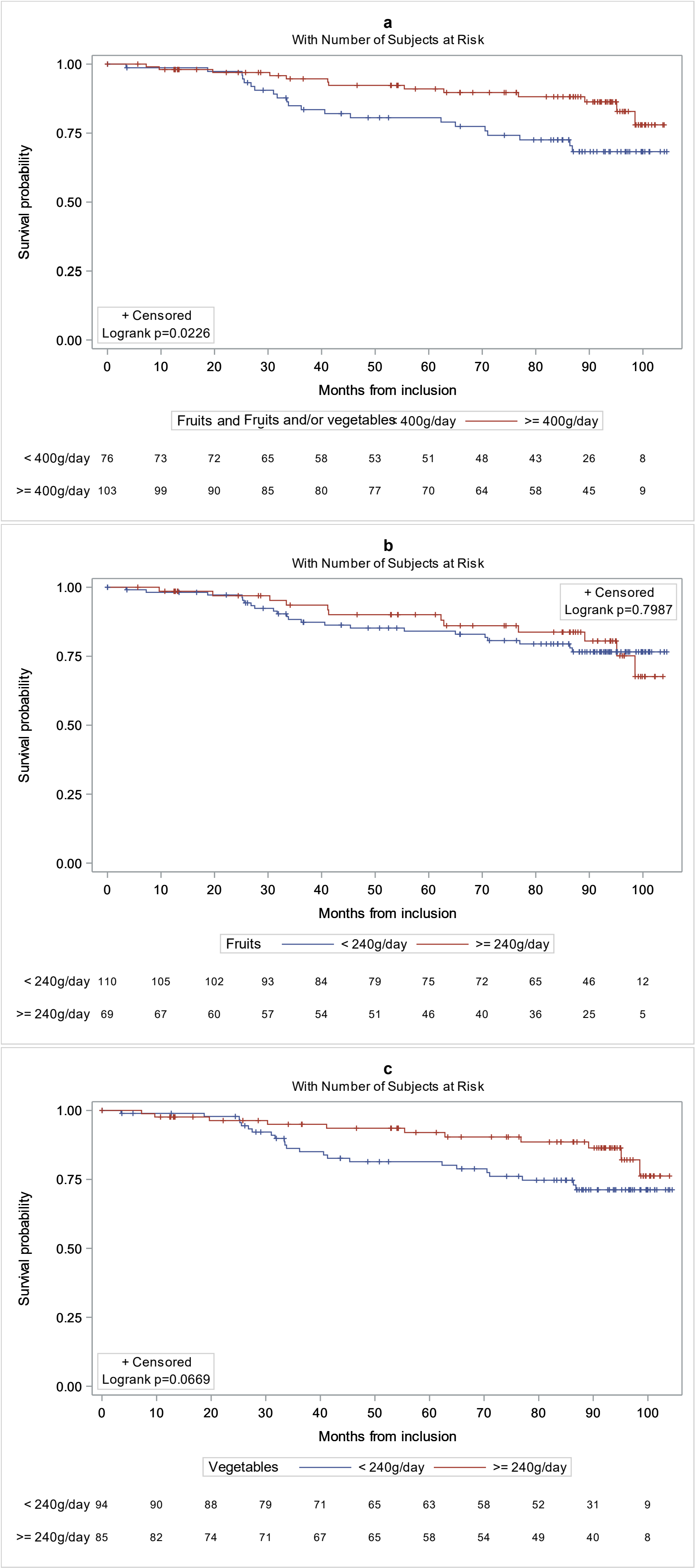
a
With Number of Subjects at Risk
Survival probability
1.00
0.75
0.50
0.25
0.00
+ Censored
Logrank p=0.0226
0 10 20 30 40 50 60 70 80 90 100
Months from inclusion
Fruits and Fruits and/or vegetables < 400g/day >= 400g/day
< 400g/day 76 73 72 65 58 53 51 48 43 26 8
>= 400g/day 103 99 90 85 80 77 70 64 58 45 9
b
With Number of Subjects at Risk
Survival probability
1.00
0.75
0.50
0.25
0.00
+ Censored
Logrank p=0.7987
0 10 20 30 40 50 60 70 80 90 100
Months from inclusion
Fruits < 240g/day >= 240g/day
< 240g/day 110 105 102 93 84 79 75 72 65 46 12
>= 240g/day 69 67 60 57 54 51 46 40 36 25 5
c
With Number of Subjects at Risk
Survival probability
1.00
0.75
0.50
0.25
0.00
+ Censored
Logrank p=0.0669
0 10 20 30 40 50 60 70 80 90 100
Months from inclusion
Vegetables < 240g/day >= 240g/day
< 240g/day 94 90 88 79 71 65 63 58 52 31 9
>= 240g/day 85 82 74 71 67 65 58 54 49 40 8

Fig. S2. Kaplan-Meier curves for incident HCC or death related to a liver disease among patients with liver cirrhosis who consumed a) fruit and/or vegetable, b) fruit, and c) vegetable at or above thresholds (n=179). Level of significance: p= 0.05 (logrank test).

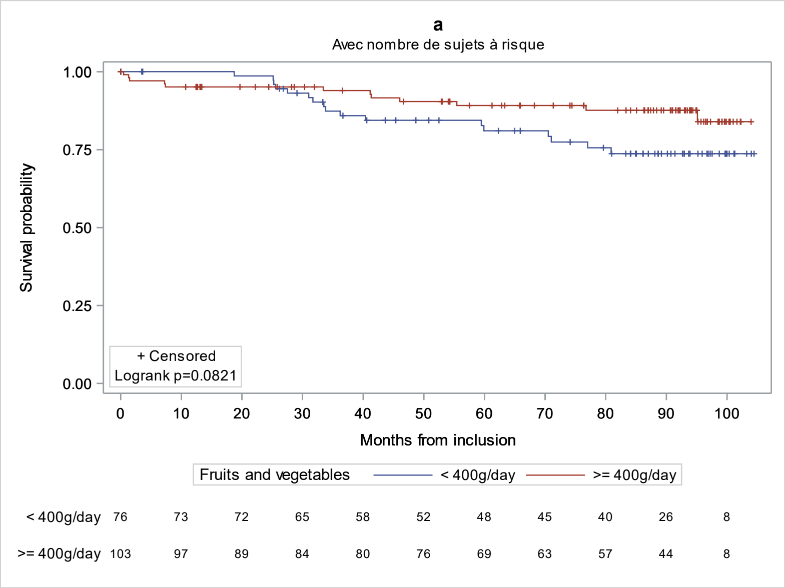
a
Avec nombre de sujets à risque
Survival probability
1.00
0.75
0.50
0.25
0.00
+ Censored
Logrank p=0.0821
0 10 20 30 40 50 60 70 80 90 100
Months from inclusion
Fruits and vegetables
< 400g/day
>= 400g/day
< 400g/day 76 73 72 65 58 52 48 45 40 26 8
>= 400g/day 103 97 89 84 80 76 69 63 57 44 8

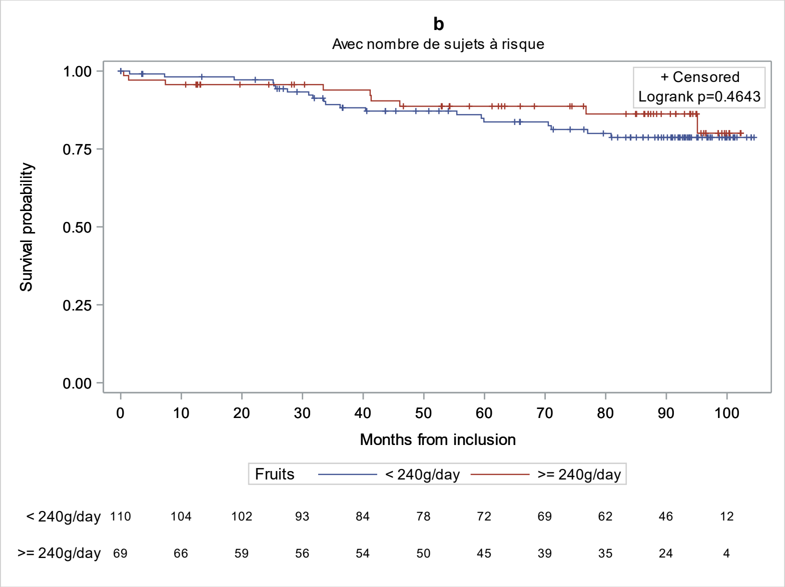
b
Avec nombre de sujets à risque
Survival probability
1.00
0.75
0.50
0.25
0.00
+ Censored
Logrank p=0.4643
0 10 20 30 40 50 60 70 80 90 100
Months from inclusion
Fruits
< 240g/day
>= 240g/day
< 240g/day 110 104 102 93 84 78 72 69 62 46 12
>= 240g/day 69 66 59 56 54 50 45 39 35 24 4

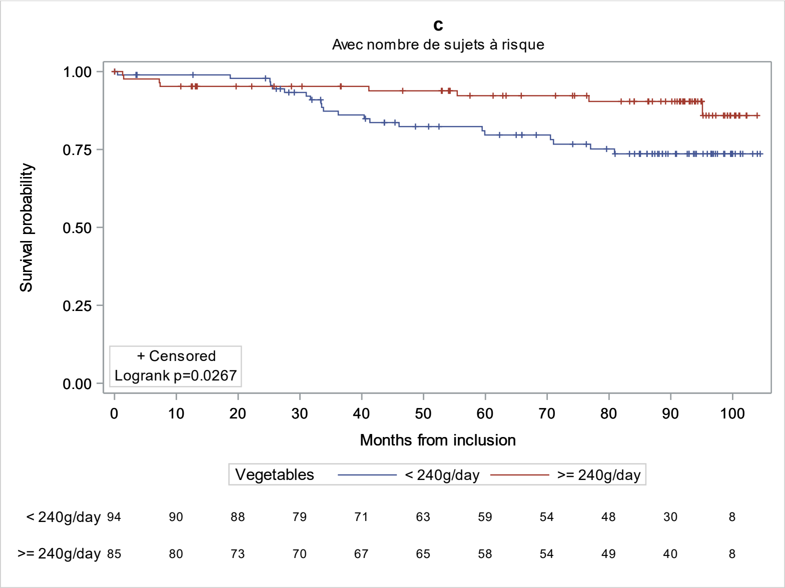
c
Avec nombre de sujets à risque
Survival probability
1.00
0.75
0.50
0.25
0.00
+ Censored
Logrank p=0.0267
0 10 20 30 40 50 60 70 80 90 100
Months from inclusion
Vegetables
< 240g/day
>= 240g/day
< 240g/day 94 90 88 79 71 63 59 54 48 30 8
>= 240g/day 85 80 73 70 67 65 58 54 49 40 8

Fig. S3. Kaplan-Meier curves for incident all hepatic events (HCC and decompensation defined according to BAVENO VII) among patients with liver cirrhosis who consumed a) fruit and/or vegetable, b) fruit, and c) vegetable at or above thresholds (n=179). Level of significance: p= 0.05 (logrank test).

Table S1. Comparisons of patients' baseline characteristics according to incident HCC (n= 179). Level of significance: p= 0.05 (Chi-squared test or Fisher's exact test for categorical variables and Wilcoxon rank-sum test the quantitative variable).

		%		
		**HCC+ (n= 20)**	**HCC- (n= 159)**	**p-value**
Sex	Male	80.0	69.8	0.34
	Female	20.0	30.2	
Age (years)	<50	5.0	17.0	0.05
	[50-60[	35.0	35.2	
	[60-70[	55.0	28.9	
	>= 70	5.0	18.9	
Body mass index (kg/m²)	<25	40.0	37.1	0.92
	[25-30[	40.0	39.0	
	≥30	20.0	23.9	
Education level	No high school diploma	35.0	39.6	0.73
	High school diploma	50.0	40.9	
	University degree	15.0	19.5	
Marital status	Single	25.0	33.3	0.45
	Cohabiting	75.0	66.7	
Occupational status	Employed	25.0	39.6	0.35
	Not in the labor force	60.0	52.2	
	Sick leave	15.0	8.2	
Smoking status	Former smoker or non-smoker	55.0	74.8	0.06
	Smoker	45.0	25.2	
Alcohol consumption	Never	65.0	59.1	0.50
	Occasionally	15.0	26.4	
	Regularly	20.0	14.5	
Level of total physical activity	High	15.0	13.8	0.15
	Moderate	30.0	52.8	
	Low	45.0	23.9	
	Missing	10.0	9.4	
Liver cirrhosis causes	Alcoholic	60.0	42.1	0.13
	Viral	40.0	57.9	
Country or region of birth	Africa	10.0	16.5	0.09
	Asia	10.0	10.8	
	Europe	5.0	14.6	
	France	75.0	44.0	
	Maghreb	0.0	14.6	
Coffee consumption (g/day)	0	15.0	23.3	0.68
	]1-93[	25.0	25.2	
	≥93	60.0	51.6	
Dietary energy (kcal)	Median (Q1-Q3)	1890.3 (1499.6-2487.4)	1933.6 (1286.5-2626.1)	0.83
History of diabetes	No	70.0	71.1	0.92
	Yes	30.0	28.9	

p-values were obtained using Chi-squared test or Fisher's exact test for categorical variables and Wilcoxon rank-sum test for the quantitative variable.

Table S2. Comparisons of patients' baseline characteristics according to fruit consumption (n= 179). Level of significance: p= 0.05 (Chi-squared test or Fisher's exact test for categorical variables and Wilcoxon rank-sum test the quantitative variable).

		%		
		**<240 g/day (n= 110)**	**>= 240 g/day (n= 69)**	**p-value**
Sex	Male	69.1	73.9	0.49
	Female	30.9	26.1	
Age (years)	<50	14.5	17.3	0.65
	[50-60[	38.2	30.4	
	[60-70[	28.2	37.7	
	>= 70	19.0	14.4	
Body mass index (kg/m²)	<25	40.0	33.3	0.44
	[25-30[	35.4	44.9	
	≥30	24.5	21.7	
Education level	No high school diploma	38.2	40.6	0.83
	High school diploma	43.6	39.1	
	University degree	18.2	20.3	
Marital status	Single	32.7	31.9	0.91
	Cohabiting	67.3	68.1	
Occupational status	Employed	35.4	42.0	0.42
	Not in the labor force	53.6	52.2	
	Sick leave	10.9	5.8	
Smoking status	Former smoker or non-smoker	66.4	82.6	0.02
	Smoker	33.6	17.4	
Alcohol consumption	Never	61.8	56.5	0.77
	Occasionally	23.6	27.5	
	Regularly	14.5	15.9	
Level of total physical activity	High	10.0	20.3	0.24
	Moderate	50.9	49.3	
	Low	29.1	21.7	
	Missing	10.0	8.7	
Liver cirrhosis causes	Alcoholic	42.7	46.4	0.63
	Viral	57.3	53.6	
Country or region of birth	Africa	15.4	15.9	0.01
	Asia	10.0	11.6	
	Europe	8.2	21.7	
	France	56.4	33.3	
	Maghreb	10.0	17.4	
Coffee consumption (g/day)	0	22.7	21.7	0.84
	]1-90[	23.6	27.5	
	≥90	53.6	50.7	
Dietary energy (kcal)	Median (Q1-Q3)	1768.5 (1339.4-2360.6)	2239.3 (1744.2-2772.8)	0.002
History of diabetes	No	71.8	69.6	0.75
	Yes	28.2	30.4	

p-values were obtained using Chi-squared test or Fisher's exact test for categorical variables and Wilcoxon rank-sum test the quantitative variable.

Table S3. Comparisons of patients' baseline characteristics according to vegetable consumption (n= 179). Level of significance: p= 0.05 (Chi-squared test or Fisher's exact test for categorical variables and Wilcoxon rank-sum test the quantitative variable).

		%		
		<240 g/day (n= 94)	>= 240 g/day (n= 85)	p-value
Sex	Male	70.2	71.8	0.82
	Female	29.8	28.2	
Age (years)	<50	12.7	18.8	0.33
	[50-60[	40.4	29.4	
	[60-70[	27.7	36.5	
	>= 70	19.2	15.3	
Body mass index (kg/m²)	<25	36.2	38.8	0.79
	[25-30[	38.3	40.0	
	≥30	25.5	21.2	
Education level	No high school diploma	38.3	40.0	0.88
	High school diploma	43.6	40.0	
	University degree	18.1	20.0	
Marital status	Single	31.9	32.9	0.88
	Cohabiting	68.1	67.1	
Occupational status	Employed	37.2	38.6	0.94
	Not in the labor force	53.2	52.9	
	Sick leave	9.6	8.2	
Smoking status	Former smoker or non-smoker	68.1	77.6	0.15
	Smoker	31.9	22.3	
Alcohol consumption	Never	57.4	62.3	0.71
	Occasionally	25.5	24.7	
	Regularly	17.0	12.9	
Level of total physical activity	High	8.5	20.0	0.08
	Moderate	50.0	50.6	
	Low	31.9	20.0	
	Missing	9.6	9.4	
Liver cirrhosis causes	Alcoholic	46.8	41.2	0.45
	Viral	53.2	58.8	
Country or region of birth	Africa	16.0	15.3	0.40
	Asia	7.4	14.1	
	Europe	12.8	14.1	
	France	53.2	41.2	
	Maghreb	10.6	15.3	
Coffee consumption (g/day)	0	26.6	17.6	0.22
	]1-90[	26.6	23.5	
	≥90	46.8	58.8	
Dietary energy (kcal)	Median (Q1-Q3)	1890.3 (1499.6-2487.4)	2138.1 (1709.4-2772.8)	0.001
History of diabetes	No	72.3	69.4%	0.67
	Yes	27.7	30.6%	

p-values were obtained using Chi-squared test or Fisher's exact test for categorical variables and Wilcoxon rank-sum test for the quantitative variable.

Table S4. Standardized differences in confounders between patients below the fruit and/or vegetable consumption threshold and patients above or at the fruit and/or vegetable consumption threshold before and after the use of the propensity score

Cofounder	Fruit and/or vegetable consumption		Fruit consumption		Vegetable consumption	
	Standardized difference before propensity score	Standardized difference after propensity score	Standardized difference before propensity score	Standardized difference after propensity score	Standardized difference before propensity score	Standardized difference after propensity score
Dietary energy	0.37732	0.07157	0.38451	0.08698	0.46601	0.03458
Sex	0.14682	-0.00425	0.10698	0.01798	0.03420	0.04769
Education level	0.14634	0.06625	0.10215	0.18309	0.08338	0.04200
Occupational status	0.14869	0.08089	0.20823	0.27564	0.07419	0.03703
Smoking status	-0.37024	-0.11248	-0.37929	0.09521	-0.21630	-0.15997
Level of physical activity	0.41905	0.05837	0.29950	0.12910	0.37952	0.09919
Liver cirrhosis causes	0.24488	0.05230	0.29004	-0.07570	0.33316	-0.00798
Country or region of birth	0.58106	0.12689	0.54822	0.17632	0.31564	0.12591
Alcohol consumption	0.08711	0.03366	0.10524	0.08585	0.12377	0.03301
Body mass index	0.12274	0.02317	0.20665	0.06267	0.11880	0.06549
Age	0.50918	0.22750	0.29332	0.11682	0.35780	0.07760
History of diabetes	-0.12511	-0.04817	-0.02910	-0.03267	-0.07467	-0.03966

Cofounder	Fruit and/or vegetable consumption		Fruit consumption		Vegetable consumption	
	Standardized difference before propensity score	Standardized difference after propensity score	Standardized difference before propensity score	Standardized difference after propensity score	Standardized difference before propensity score	Standardized difference after propensity score
Coffee consumption	0.17707	0.04795	0.09145	0.12184	0.26148	0.04922

Table S5. Associations between fruit and/or vegetable consumption and incident all hepatic events (HCC and decompensation defined according to BAVENO VII) (n= 179). Level of significance: p= 0.05 (unadjusted [Model 1] and adjusted Poisson regression models [Model 2]).

	Model 1[a]			Model 2[b]		
	RR	95% CI	p	RR	95% CI	p
Fruit and/or vegetable consumption						
<400 g/day (n= 76)	1.00			1.00		
≥400 g/day (n=103)	0.52	[0.25- 1.08]	0.08	0.72	[0.34- 1.54]	0.39
Fruit consumption						
<240 g/day (n= 110)	1.00			1.00		
≥240 g/day (n= 69)	0.75	[0.34- 1.64]	0.47	1.03	[0.46- 2.34]	0.94
Vegetable consumption						
<240 g/day (n= 94)	1.00			1.00		
≥240 g/day (n= 85)	**0.40**	**[0.18- 0.91]**	**0.03**	0.49	[0.22- 1.08]	0.08

Abbreviations: RR, relative risk; 95% CI, 95% confidence interval.

[a] *Unadjusted Poisson regression models.*

[b] *Poisson regression models adjusted on dietary energy, age, sex, liver cirrhosis causes, history of diabetes, smoking status, alcohol consumption, body mass index, level of physical activity, coffee consumption, education level, occupational status, country or region of birth using inverse probability of treatment weighting with propensity scores.*

Results for which the 95% CI excludes the null are bolded

Table S6. Associations between fruit and/or vegetable consumption and incident HCC: unadjusted and adjusted Cox regression models (n= 179). Level of significance: p= 0.05 (unadjusted [Model 1] and adjusted Cox regression models [Model 2]).

	Model 1[a]			Model 2[b]		
	HR	95% CI	p	HR	95% CI	p
Fruit and/or vegetable consumption						
<400 g/day (n= 76)	1.00			1.00		
≥400 g/day (n=103)	0.41	[0.16- 1.02]	0.055	0.50	[0.18- 1.36]	0.17
Fruit consumption						
<240 g/day (n= 110)	1.00			1.00		
≥240 g/day (n= 69)	0.56	[0.20- 1.53]	0.26	0.87	[0.30- 2.53]	0.80
Vegetable consumption						
<240 g/day (n= 94)	1.00			1.00		
≥240 g/day (n= 85)	0.37	[0.13- 1.01]	0.052	0.36	[0.13- 1.00]	0.051

Abbreviations: HR, hazard ratio; 95% CI, 95% confidence interval.

[a] *Unadjusted Cox regression models.*

[b] *Cox regression models adjusted on dietary energy, age, sex, liver cirrhosis causes, history of diabetes, smoking status, alcohol consumption, body mass index, level of physical activity, coffee consumption, education level, occupational status, country or region of birth using inverse probability of treatment weighting with propensity scores.*

Results for which the 95% CI excludes the null are bolded.

Table S7. Associations between fruit and/or vegetable consumption and incident HCC or death related to a liver disease (n= 179). Level of significance: p= 0.05 (unadjusted [Model 1] and adjusted Cox regression models [Model 2]).

	Model 1[a]			Model 2[b]		
	HR	95% CI	p	HR	95% CI	p
Fruit and/or vegetable consumption						
<400 g/day (n= 76)	1.00			1.00		
≥400 g/day (n=103)	**0.46**	**[0.23- 0.91]**	**0.03**	0.63	[0.31- 1.30]	0.21
Fruit consumption						
<240 g/day (n= 110)	1.00			1.00		
≥240 g/day (n= 69)	0.91	[0.45- 1.84]	0.80	1.55	[0.74- 3.25]	0.25
Vegetable consumption						
<240 g/day (n= 94)	1.00			1.00		
≥240 g/day (n= 85)	0.52	[0.25- 1.06]	0.07	0.63	[0.31- 1.26]	0.19

Abbreviations: HR, hazard ratio; 95% CI, 95% confidence interval.

[a] *Unadjusted Cox regression models.*

[b] *Cox regression models adjusted on dietary energy, age, sex, liver cirrhosis causes, history of diabetes, smoking status, alcohol consumption, body mass index, level of physical activity, coffee consumption, education level, occupational status, country or region of birth using inverse probability of treatment weighting with propensity scores.*

Results for which the 95% CI excludes the null are bolded

Table S8. Associations between fruit and/or vegetable consumption and incident all hepatic events (HCC and decompensation defined according to BAVENO VII) (n= 179). Level of significance: p= 0.05 (unadjusted [Model 1] and adjusted Cox regression models [Model 2]).

	Model 1[a]			Model 2[b]		
	HR	95% CI	p	HR	95% CI	p
Fruit and/or vegetable consumption						
<400 g/day (n= 76)	1.00			1.00		
≥400 g/day (n=103)	0.52	[0.25- 1.10]	0.09	0.72	[0.34- 1.54]	0.40
Fruit consumption						
<240 g/day (n= 110)	1.00			1.00		
≥240 g/day (n= 69)	0.75	[0.34- 1.64]	0.46	1.06	[0.46- 2.41]	0.89
Vegetable consumption						
<240 g/day (n= 94)	1.00			1.00		
≥240 g/day (n= 85)	**0.41**	**[0.18- 0.93]**	**0.03**	0.50	[0.23- 1.09]	0.08

Abbreviations: HR, hazard ratio; 95% CI, 95% confidence interval.

[a] *Unadjusted Cox regression models.*

[b] *Cox regression models adjusted on dietary energy, age, sex, liver cirrhosis causes, history of diabetes, smoking status, alcohol consumption, body mass index, level of physical activity, coffee consumption, education level, occupational status, country or region of birth using inverse probability of treatment weighting with propensity scores.*

Results for which the 95% CI excludes the null are bolded.
