# Supplementary material for: Associations between fruit and vegetable consumption and HCC occurrence in patients with cirrhosis
Source: JHEP Rep. 2025 Feb 13;7(5):101355. doi: 10.1016/j.jhepr.2025.101355 (PMC12008579; doi:10.1016/j.jhepr.2025.101355)
Supplement: Multimedia component 4 [file mmc4.pdf]

Research article

JHEP|Reports

Associations between fruit and vegetable consumption and HCC occurrence in patients with cirrhosis

Authors

Florian Manneville, Zineb Zouakia, Séverine Donneger, …, Mathilde Touvier, Nathalie Ganne-Carrié, Chantal Julia

Correspondence

florian.manneville@eren.smbh.univ-paris13.fr (F. Manneville).

Graphical abstract

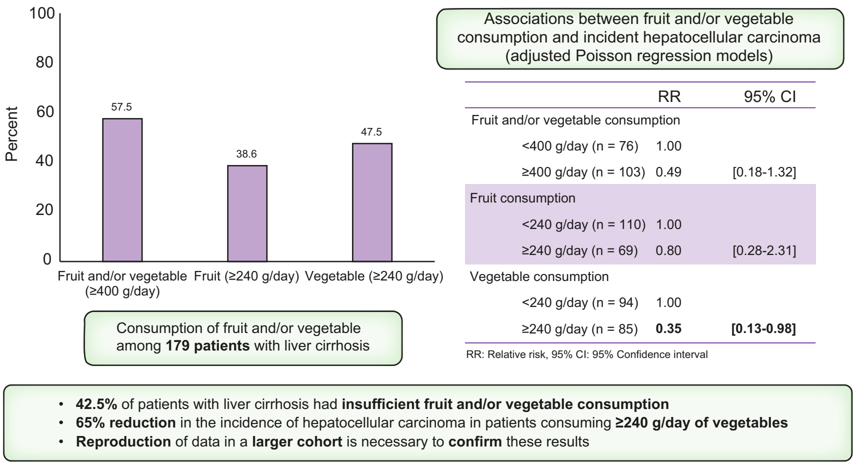

Highlights:

- A total of 42.5% of patients with cirrhosis had insufficient fruit and/or vegetable consumption.
- There was no evidence of an association between fruit consumption and hepatocellular carcinoma.
- A 65% reduction in the incidence of hepatocellular carcinoma was observed in patients consuming ≥240 g/day of vegetables.

Impact and implications:

The association between fruit and vegetable consumption and the risk of hepatocellular carcinoma (HCC) is poorly documented in the population of patients with cirrhosis, while such knowledge is crucial for adapting HCC prevention messages. Our study shows 57.5% of patients with cirrhosis reported consuming fruit and/or vegetables at or above the French and WHO threshold of 400 g/day, with a higher proportion of patients consuming at least 240 g/day of vegetables compared with those consuming at least 240 g/day of fruit (47.5% *vs.* 38.6%). The results suggest that consuming at least 240 g/day of vegetables reduces the risk of HCC by 65% in patients with cirrhosis.

https://doi.org/10.1016/j.jhepr.2025.101355

© 2025 The Author(s). Published by Elsevier B.V. on behalf of European Association for the Study of the Liver (EASL). This is an open access article under the CC BY license (http://creativecommons.org/licenses/by/4.0/). JHEP Reports, 2025, 7, 1–9

Associations between fruit and vegetable consumption and HCC occurrence in patients with cirrhosis

Florian Manneville[1,*], Zineb Zouakia[1], Séverine Donneger[2], Leopold K. Fezeu[1], Alice Bellicha[1], Pierre Nahon[2,3,4], Mathilde Touvier[1], Nathalie Ganne-Carrié[2,3,4,†], Chantal Julia[1,5,†]

JHEP Reports **2025**. vol. 7 | 1–9

Check for updates

Background & Aims: Prospective studies are needed to increase knowledge of fruit and vegetable consumption effects on hepatocellular carcinoma (HCC) risk. This study aimed to investigate the association between fruit and vegetable consumption and incident HCC in French patients with cirrhosis.

Methods: This study used data from a French prospective observational cohort nested in two national prospective cohorts of patients with histologically proven compensated alcohol-related or viral cirrhosis. Fruit and vegetable consumption was assessed by a trained dietitian using a semiquantitative food-frequency questionnaire validated in French and analyzed as binary exposure according to predefined thresholds (≥240 g/day for fruit or vegetables and ≥400 g/day for fruit and vegetables combined). Incident HCC was primary outcome. Propensity scores were used in Poisson regression models.

Results: Among 179 patients analyzed, 20 HCC were diagnosed during follow-up (median 7.3 [Q1–Q3: 4.1–8.0] years). A significant association was observed between HCC incidence and vegetable consumption ≥240 g/day (adjusted relative risk [RR] 0.35, 95%CI [0.13; 0.98], p = 0.04), but not with consumption of fruit and vegetable ≥400 g/day (RR = 0.49, 95%CI [0.18; 1.32], p = 0.16), nor with fruit consumption ≥240 g/day (RR = 0.80, 95% CI [0.28; 2.31], p = 0.68).

Conclusions: This longitudinal study documented insufficient fruit and/or vegetable consumption in 42.5% of patients with cirrhosis and a 65% reduction of HCC incidence in those with vegetable consumption ≥240 g/day. Reproduction of results in a larger sample are necessary to explore the potential of fruit and vegetables as protective factors in HCC.

© 2025 The Author(s). Published by Elsevier B.V. on behalf of European Association for the Study of the Liver (EASL). This is an open access article under the CC BY license (http://creativecommons.org/licenses/by/4.0/).

Introduction

Liver cancer is the sixth most frequent cancer worldwide, mainly represented by hepatocellular carcinoma (HCC) (85–90%). The main etiologies of underlying chronic liver disease in Western countries are alcohol and viral hepatitis.[1,2]

Among HCC risk factors, the recent World Cancer Research Fund report has identified several modifiable lifestyle factors.[3] For example, there is strong evidence that overweight and obesity, alcohol consumption, and consumption of foods contaminated with aflatoxins (*i.e.* toxins produced by molds) significantly increase the risk of HCC. Conversely, coffee consumption and physical activity have been reported as protective factors.[3–6]

However, results of this report regarding the effects of fruit and vegetable consumption on liver cancer according to the World Cancer Research Fund.[3] In a review by George *et al.*[7] investigating the association between diet and HCC, conclusions of studies were also contradictory. For example, in a large European cohort study including healthy men and women, increase in vegetable intake was associated with a significant reduction in the risk of HCC.[8] Conversely, in a case-control study, there was no evidence of an association between HCC and vegetable intake among Greek patients without cirrhosis;[9] and an American cohort study found no significant association between vegetable fiber intake and risk of HCC.[10] With regard to fruit consumption, none of the studies included in the review by George *et al.*[7] provided evidence of an association with HCC occurrence. More recently, two American cohort studies reported no association between fruit intake and HCC risk.[10,11] With regards to HCC mortality, a cohort study conducted in Japan provided no evidence of an association between fruit and vegetable consumption and mortality in patients with a history of liver disease.[12] Finally, in the large cohort study reported by Zhao *et al.*,[11] vegetable intake was associated with a lower risk of liver cancer disease mortality, but no association was demonstrated with fruit intake.

In light of the above literature, and in line with the conclusion of George *et al.*'s review, further prospective studies are needed to increase knowledge regarding the effects of fruit and vegetable consumption on the risk of HCC.[7,13] In addition, the

* Corresponding author. Address: Equipe de Recherche en Epidémiologie Nutritionnelle (EREN), 74 rue Marcel Cachin, F-93017 Bobigny Cedex, France. Tel.: +33643263849.
E-mail address: florian.manneville@eren.smbh.univ-paris13.fr (F. Manneville).
† Equal contribution.
https://doi.org/10.1016/j.jhepr.2025.101355

majority of studies investigating this association included participants without cirrhosis. The association between fruit and vegetable consumption and HCC risk is therefore poorly documented in the population of patients with cirrhosis, while such knowledge is crucial to adapt HCC prevention messages. Also, relatively few of the aforementioned studies were conducted in Western Europe, where fruit and/or vegetable consumption and the incidence of HCC might be different from other parts of the world.[7] Therefore, this paper presents the results of a cohort study which aimed to investigate the association between fruit and/or vegetable consumption and (1) incident HCC (primary objective), (2) incident HCC or death related to a liver disease (secondary objective) in French patients with cirrhosis.

Patients and methods

Reporting of this study follows the STROBE checklist[14] (see Supplementary material). CTAT information is presented in the Supplementary CTAT Table.

Study design and setting

This study used data from the ALICIR (ALImentation and CIRrhosis) project, a French prospective observational cohort nested in two national ongoing prospective cohorts (ANRS CO12 CirVir [Complications and competing risks of death in compensated viral cirrhosis] and INCa-CIRRAL [Hepatocellular Carcinoma in Patients With Uncomplicated Alcoholic Cirrhosis: Incidence and Predictive Factors. A Multicentric Prospective Cohort]). The aim of the ALICIR study was to assess the association between dietary behavior, lifestyle (including physical activity), and environmental factors and the development of HCC in patients with viral or alcoholic cirrhosis. The ANRS CO12 CirVir cohort included 1,671 adult patients with histologically proven compensated viral cirrhosis from 35 French clinical centers, dedicated to liver diseases, and recruited between March 2006 and December 2012. This study has been described in detail elsewhere.[15] The INCa-CIRRAL cohort included adult patients with histologically proven compensated alcohol-related cirrhosis, with or without HIV co-infection, but without HBV or HCV infection, from 22 French clinical centers dedicated to liver diseases, and recruited between 2010 and 2016. The INCa-CIRRAL study was registered on ClinicalTrials.gov (No. NCT01213927) and was fully described elsewhere.[16] In both the ANRS CO12 CirVir and INCa-CIRRAL cohorts, in line with French and international guidelines on HCC screening in high risk-patients, a Doppler ultrasonography examination was performed every 6 months. The ALICIR study included patients enrolled in ANRS CO12 CirVir or INCa-CIRRAL between June 2014 and February 2016 in two tertiary liver centers in the same region (northeastern suburbs of Paris) (see Fig. 1 in Buscail *et al.*[13]). Inclusion in the ALICIR cohort occurred during an inclusion or follow-up visit to the INCa-CIRRAL and/or ANRS CO12 CirVir cohorts.

The ANRS CO12 CirVir and INCa-CIRRAL cohorts obtained approval from the ethics committee (Comité de Protection des Personnes, Aulnay-sous-Bois, France). The ALICIR study was approved by the French Advisory Committee for Data Processing in Health Research of the French Ministry of Health and Medical Research (CCTIRS) (no. 13.501) and the Commission Nationale de l'Informatique et des Libertés (CNIL) (no. DR-2014-219). All research was conducted in accordance with both the Declarations of Helsinki and Istanbul. Free and informed consent was obtained from patients during the inclusion visit. Written informed consent was obtained from participants to participate in the study.

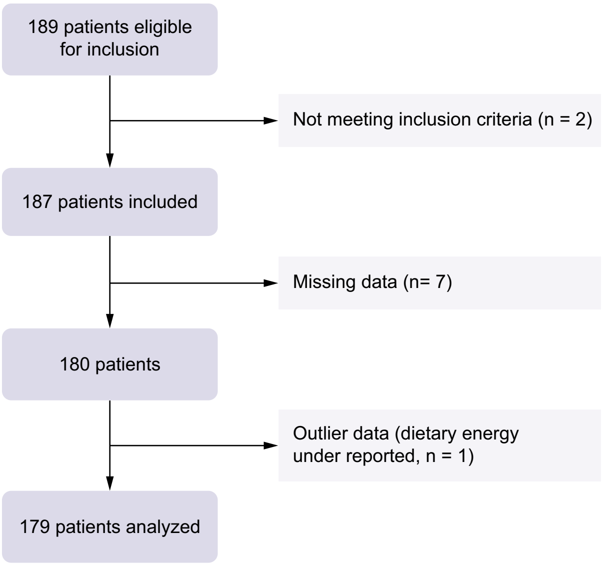

Fig. 1. Flowchart of the study.

Study population

To be included in the ALICIR cohort, patients had to be enrolled in the ANRS CO12 CirVir or INCa-CIRRAL cohorts, have no focal lesion(s) suggestive of HCC or HCC confirmed by imaging (ultrasound, magnetic resonance imaging, or computed tomography) <90 days before inclusion, and sign a free and informed consent. Patients who were not enrolled in a social security program (beneficiary or non-beneficiary), and/or who had difficulty understanding French, and/or who had a Child-Pugh score >7 (class B or C), and/or who had presented an episode of hepatic decompensation during the timeframe elapsing since CIRRAL or CirVir enrollment, were not included in the ALICIR cohort.

Measurements

Outcomes

There were three outcomes in this study. Incident HCC was the primary outcome. In case of a focal liver lesion detected by ultrasonography: (i) echogenicity, number, and diameter of lesion(s) (classified as <10 mm, 11–20 mm, 21–30 mm, 31–50 mm, or >50 mm), as well as anatomic localization according to the Couinaud classification were reported; (ii) portal vasculature (main trunk and branches), hepatic veins, and vena cava were systematically examined; (iii) a diagnostic procedure using contrast-enhanced imaging (CT-scan or MRI), serum alpha-fetoprotein assay or a guided biopsy was performed according to the 2005[17] AASLD guidelines updated in 2011.[18] The secondary outcomes were (1) incident HCC, or related liver deaths attributable to either liver failure, bleeding as a result of portal hypertension, or bacterial infections, and (2)

incident HCC or decompensation defined according to BAVENO VII[19] (*i.e.* ascites and/or encephalopathy and/or bleeding from gastro-esophageal varices). There were not enough cases of death related to liver disease in our sample to consider this outcome alone (*i.e.* without incident HCC). Information recorded during follow-up were retrieved by a dedicated clinical research associate.

During the inclusion visit, patients underwent a 1-h face-to-face interview with a trained dietitian to complete a food-frequency questionnaire validated in the French language[20] and adapted for patients with cirrhosis.

For 240 individual food items, patients were asked to declare their frequency and portion size consumption using a semiquantitative food-frequency questionnaire.[20] Frequency of consumption referred to usual consumption over the past year on an increasing scale including daily, weekly, monthly, or yearly units, as appropriate. Portion size consumption was estimated using a set of three validated color photographs showing different portion sizes.[21] Together with the two intermediate and two extreme sizes, patients had to choose one of the seven portion sizes. Daily fruit and/or vegetable consumptions (g/day) were then derived from these data. Consumptions of fruit and/or vegetable were dichotomized according to French and WHO threshold (<400 g/day or ≥400 g/day).[22,23] The threshold is equivalent to five 80-g servings of fruit and/or vegetables per day. Because the recommendation is for five servings of fruits and vegetables per day, we considered a lower threshold for vegetable-only consumption and a lower threshold for fruit-only consumption, both of which are three 80-g servings per day (*i.e.* <240 g/day or ≥240 g/day). This threshold would result in 480 g/day of fruit and vegetable consumption and was chosen because it is the lowest threshold that would allow reaching the French and WHO threshold of 400 g/day for total fruit and/or vegetable consumption per day (a threshold of two 80-g servings per day of fruit in addition to two 80-g servings per day of vegetables would result in 320 g/day, which is less than 400 g/day). Patients did not receive any dietary counseling before completing the questionnaire or during the follow-up, unless they required specific dietary counseling related to other chronic diseases such as diabetes, obesity, or heart failure.

Patient characteristics

At inclusion in the ALICIR study, patients completed self-reported questionnaires on sociodemographic data including age (years) categorized into four classes (<50, 50–60, 60–70, ≥70), sex (male, female), marital status (single, cohabiting), education level (no high school diploma, high school diploma, university degree), occupational status (employed, not in the labor force, sick leave), country or region of birth (Africa, Asia, Europe, France, Maghreb). The latter was collected to account for exposure to carcinogenic environmental agents such as aflatoxin B1. Participants self-reported smoking status (former smoker or non-smoker, smoker), current alcohol consumption (never, occasionally, regularly), body mass index (kg/m^2) categorized into three classes (<25, 25–30, ≥30),[24] and coffee consumption (g/day) categorized in three classes based on the median value observed in our sample (0, 1–90, ≥90). The International Physical Activity Questionnaire short form was used to calculate patients' level of total physical activity.[25] It is a validated questionnaire with acceptable measurement properties in which patients declared the frequency and duration of vigorous and moderate physical activity, and walking in the last 7 days.[25] The total physical activity was derived from these data, and then categorized into three levels of total physical activity according to the IPAQ scoring guidelines (high, moderate, low).[26] History of diabetes (yes, no [whatever the type of diabetes]), cirrhosis etiologies (viral [subdivided into HBV, HCV, HBV/HCV] or alcoholic), Child-Pugh score (class A or B7), metabolic dysfunction-associated steatotic liver disease [MASLD][27] (yes, no), and arterial hypertension (yes, no) were assessed by physicians as part of the ANRS CO12 CirVir and INCa-CIRRAL cohorts, and used in the ALICIR cohort.

Statistical analyses

Descriptive analyses

The repartition of participants was described using a flowchart. Fruit and/or vegetable consumption, fruit consumption, vegetable consumption, and patients' characteristics were described in numbers and percentages for categorical variables and median (Q1–Q3) for quantitative variables. Missing data were reported. The median (Q1–Q3) follow-up (years) time was calculated as the difference between the date of last follow-up or outcome and the date of inclusion. Loss to follow-up was censored at the time of the last follow-up.

Associations between fruit and/or vegetable consumption, and incident HCC

Poisson regression models were computed to investigate unadjusted (Models 1) and adjusted (Models 2) associations between fruit and/or vegetable consumption and incident HCC. Models 2 were adjusted for patient characteristics that could be confounders based on the literature, and on characteristics that were associated with both fruit and/or vegetable consumptions and outcomes (X^2 test or Fisher's exact test and Wilcoxon rank-sum test (Table 1, Tables S1–S3). Given the large number of patients' characteristics that required to be included in the adjusted model and the relatively small number of occurrences of HCC,[28] a propensity score was calculated. The propensity score was defined as the probability for each patient to have a fruit and/or vegetable consumption superior or equal to the French and WHO thresholds, conditional on his/her observed characteristics.[29] Computation of a propensity score allows to design and analyze an observational study so that it mimics some of the particular characteristics of a randomized controlled trial, especially the equal distribution of known confounding factors between groups.[29] Three propensity scores were calculated regressing potential confounders (*i.e.* dietary energy, age, sex, cirrhosis causes, history of diabetes, smoking status, alcohol consumption, body mass index, level of physical activity, coffee consumption, education level, occupational status, country or region of birth) on fruit and/or vegetable consumption using three logistic regression models. Propensity scores were stabilized by dividing them by the mean weights. Then inverse probability of treatment weighting using the propensity scores were used to model the association between fruit and/or vegetable consumption and incident HCC using Poisson regression models (Model 2).[29,30] In such a model, patients are entered into the statistical analysis

Table 1. Comparisons of patients' baseline characteristics according to fruit and/or vegetable consumption (n = 179).

	n (%)		
	<400 g/day (n = 76)	≥400 g/day (n = 103)	*p* value
Sex			
Male	67.1	73.8	0.33
Female	32.9	26.2	
Age (years)			
<50	13.1	17.5	0.04
50–60	43.4	29.1	
60–70	22.4	38.8	
≥70	21.0	14.5	
BMI (kg/m^2)			
<25	36.8	37.9	0.72
25–30	36.8	40.8	
≥30	26.3	21.4	
Education level			
No high school diploma	35.5	41.7	0.61
High school diploma	46.0	38.8	
University degree	18.4	19.4	
Marital status			
Single	39.5	27.2	0.08
Cohabiting	60.5	72.8	
Occupational status			
Employed	35.5	39.8	0.48
Not in the labor force	52.6	53.4	
Sick leave	11.8	6.8	
Smoking status			
Former smoker or non-smoker	63.2	79.6	0.01
Smoker	36.8	20.4	
Alcohol consumption			
Never	59.2	60.2	0.79
Occasionally	23.7	26.2	
Regularly	17.1	13.6	
Level of total physical activity			
High	9.2	17.5	0.07
Moderate	46.0	53.4	
Low	35.5	19.4	
Missing	9.2	9.7	
Cirrhosis causes			
Alcoholic	50.0	39.8	0.17
Viral	50.0	60.2	
Country or region of birth			
Africa	13.2	17.5	0.008
Asia	7.9	12.6	
Europe	7.9	17.5	
France	63.2	35.9	
Maghreb	7.9	16.5	
Coffee consumption (g/day)			
0	26.3	19.4	0.52
1–90	25.0	25.2	
≥90	48.7	55.3	
Dietary energy (kcal)			
Median (Q1–Q3)	1,653.4 (1,246.3–2,345.6)	2,057.3 (1,686.6–2,748.2)	0.003
History of diabetes			
No	69.7	71.8	0.76
Yes	30.3	28.2	

Level of significance: p = 0.05 (X^2 test or Fisher's exact test for categorical variables and Wilcoxon rank-sum test the quantitative variable).

according to their weight based on the propensity score, so that it accounts for measured baseline confounders.[29,30] The standardized differences in confounders between patients below the fruit and/or vegetable consumption threshold and patients above or at the fruit and/or vegetable consumption threshold were calculated before and after the use of the propensity score to assess the quality of confounding adjustment using the propensity score.[29] The differences should be closer to 0 and <0.25 after using the propensity score.[29,31] Patients who died during follow-up were censored at the date of death. Relative risks and 95% CI are reported.

Associations between fruit and/or vegetable consumption and secondary outcomes

The same Poisson regression models as described above were computed using (1) incident HCC or death related to liver disease, and (2) incident HCC or decompensation as the

outcomes. Patients who died during follow-up from non-liver disease-related causes were censored at the date of death.

Sensitivity analyses

Given the small sample size and number of events, survival analysis was performed to check for the robustness of the Poisson models, especially with respect to the strength of the associations. Kaplan–Meier curves were computed with log-rank tests. Also, three unadjusted (Model 1) and adjusted (Model 2) Cox regression models were computed to investigate the associations between fruit, vegetable, fruit and/or vegetable consumptions, and outcomes. The endpoint date for the analyses was July 17, 2023. The proportional hazard assumption was assessed using the Schoenfeld residual method. Hazard ratios (HRs) and 95% CI are reported.

We used SAS (Statistical Analysis Software 9.4, SAS Institute Inc, Cary, NC, USA) for statistical analyses. A two-sided p <0.05 was considered statistically significant.

Results

Descriptive analyses

Of the 189 patients eligible for the ALICIR study, 10 were not included in the analyses because they did not meet the inclusion criteria or had missing or outlier data (Fig. 1). A total of 179 patients were included in the analyses with a median (Q1–Q3) follow-up of 7.3 (4.1–8.0) years. At baseline, half of the patients were ≥60 years, 70.9% were male, 19.0% had a university degree, 38.0% were employed, 32.4% were single, and 52.5% were not born in France. Overall, 27.4% were smokers, 15.1% were regular alcohol consumers, half had a moderate physical activity level, 57.0% had a viral cirrhosis (33.0% with HCV), 95.5% were with Child-Pugh A, and one-third had a history of diabetes (Table 2). Respectively, 57.5%, 38.6%, and 47.5% of the patients reported fruit and/or vegetable consumption, fruit consumption, and vegetable consumption at or above thresholds (*i.e.* >400 g/day for fruit or vegetable, >240 g/day for fruit, and >240 g/day for vegetable). Distributions of patients' characteristics according to fruit and vegetable consumption are shown in Table 1.

Associations between fruit and/or vegetable consumption, and incident HCC

Twenty patients (11.2% of the study sample) had incident HCC during the follow-up. Unadjusted results (Model 1) showed that consumption of ≥400 g/day of fruit and/or vegetables was associated with a borderline non-significant decrease of incident HCC by 59% (relative risk [RR] = 0.41, 95% CI [0.17; 1.03], p = 0.06) (Table 3). Results were similar for vegetable consumption ≥240 g/day (RR = 0.37, 95% CI [0.13; 1.02], p = 0.06), and non-significant for fruit consumption ≥240 g/day (RR = 0.57, 95% CI [0.21; 1.58], p = 0.28). The results were significant after adjustment using the propensity score for vegetable consumption ≥240 g/day (RR = 0.35, 95% CI [0.13; 0.98], p = 0.04), and non-significant for fruit and/or vegetable consumption ≥400 g/day (RR = 0.49, 95% CI [0.18; 1.32], p = 0.16), and fruit consumption ≥240 g/day (RR = 0.80, 95% CI [0.28; 2.31], p = 0.68) (Table 3). Standardized differences indicated acceptable differences in the prevalence of cofounders

Table 2. Baseline characteristics of the study sample (n = 179).

	n	%
Sex		
Male	127	70.9
Female	52	29.1
Age (years)		
<50	28	15.7
50–60	63	35.2
60–70	57	31.8
≥70	31	17.3
Education level		
No high school diploma	70	39.1
High school diploma	75	41.9
University degree	34	19.0
Marital status		
Single	58	32.4
Cohabiting	121	67.6
Occupational status		
Employed	68	38.0
Not in the labor force	95	53.1
Sick leave	16	8.9
Smoking status		
Former smoker or non-smoker	130	72.6
Smoker	49	27.4
Alcohol consumption		
Never	107	59.8
Occasionally	45	25.1
Regularly	27	15.1
Level of total physical activity		
High	25	14.0
Moderate	90	50.3
Low	47	26.3
Missing	17	9.5
Cirrhosis causes		
Alcohol-related	77	43.0
HBV	40	22.3
HCV	59	33.0
HBV/HCV	3	1.7
Child-Pugh score		
A	171	96.1
B7	7	3.9
BMI (kg/m^2)		
<25	67	37.6
25–30	69	38.8
≥30	42	23.6
History of diabetes		
No	102	66.7
Yes	51	33.3
Arterial hypertension		
Yes	55	30.7
No	124	69.3
MASLD*		
Yes	136	76.0
No	43	24.0
Country or region of birth		
Africa	28	15.6
Asia	19	10.6
Europe	24	13.4
France	85	47.5
Maghreb	23	12.8
Coffee consumption (g/day)		
0	40	22.3
1–90	45	25.1
≥90	94	52.5
Dietary energy (kcal)		
Median, Q1–Q3	1,890.3	1,480.5–2,550.6
Fruit and/or vegetable consumption (g/day)		
Median, Q1–Q3	469.5	288.7–709.2
<400	76	42.5

(*continued on next page*)

Table 2. (*continued*)

	n	%
≥400	103	57.5
Fruit consumption (g/day)		
Median, Q1–Q3	200.1	113.5–317.5
<240	110	61.4
≥240	69	38.6
Vegetable consumption (g/day)		
Median, Q1–Q3	234.4	155.8–386.2
<240	94	52.5
≥240	85	47.5

MASLD, metabolic dysfunction-associated steatotic liver disease.
*According to Rinella *et al.*[27]

between groups of patients for fruit and/or vegetable consumption after using propensity scores (Table S4).

Associations between fruit and/or vegetable consumption and secondary outcomes

Thirty-four patients (19.0% of the study sample) had incident HCC or death related to a liver disease. Among them, eight had incident HCC and were alive at the end of the study, eight had incident HCC and died of liver-related disease, one had incident HCC and died of non-liver-related disease, three had incident HCC and died of unknown cases, 14 had not incident HCC and died of liver-related disease. The direction of associations between fruit and/or vegetable consumption, and incident HCC or death related to a liver disease were similar to incident HCC only, but RRs were with lower effect sizes and were not statistically significant (Table 4). A total of 29 (16.2%) patients had incident HCC or decompensation. There was a trend (although not statistically significant in the adjusted analysis) for consumption of at least 240 g of vegetables per day to reduce the risk of HCC, including all hepatic events (decompensation) (Table S5).

Sensitivity analyses

Results of the Cox regression models were very close to those of Poisson regression models but not statistically significant. For example, vegetable consumption ≥240 g/day reduced the risk of HCC by 64%, but did not reach statistical significance (HR = 0.36, 95% CI [0.13; 1.00], p = 0.051) (Figs. S1–S3, Tables S6–8).

Discussion

This study suggests that consuming at least 240 g/day of vegetables reduced the risk of HCC by 65% among patients with cirrhosis. Overall, 57.5% of patients with cirrhosis reported consuming fruit and/or vegetables at or above the French and WHO threshold of 400 g/day, with a higher proportion of patients consuming at least 240 g/day of vegetable compared with those consuming at least 240 g/day of fruit (47.5% *vs.* 38.6%).

Although fruit and vegetable consumption is encouraged in France in the overall population and in specific subgroups such as in patients with cirrhosis,[32] the majority of patients in our study did not meet the French and WHO recommendations for consumption. Notably, fruit and vegetable consumption in our population could be similar to that of the healthy French population. A previous study comparing dietary intakes between ALICIR participants and French adult volunteers in a web-based cohort study did not show significant differences in the consumption of fruit and vegetables.[13] Other studies have found a lower quality of diet in patients with cirrhosis. Pashayee-Khamene *et al.*[33] showed common protein-energy malnutrition, with a tendency for low consumption of fruit and/or vegetable among patients with cirrhosis. This could relate to improper knowledge and perception of diet among patients with cirrhosis, regardless of the level of education.[34] This aligns with Volk *et al.*,[35] who showed that 47% of patients with cirrhosis had inadequate knowledge about the self-management of their disease. Another study pointed out that 20.7% of patients with cirrhosis reported an unmet need for dietary modification.[36] These results underlines the importance of appropriate nutritional education and continued monitoring for patients with cirrhosis.[37] Such an approach could be integrated into therapeutic education programs. The results of a quasi-experimental study showed that an educational intervention improved the knowledge of patients with cirrhosis including nutritional management in cirrhosis.[38]

The association between vegetable consumption and HCC risk found in our study is overall in line with the literature.[7] For example, the results of a large European cohort demonstrated a significant 17% reduction in the risk of HCC associated with a 100 g/day increase in vegetable intake.[8] This would correspond to a 40.8% reduction associated with a vegetable consumption of 240 g/day, which is 25 percentage points lower than the

Table 3. Associations between fruit and/or vegetable consumption and incidence of HCC (n = 179).

	Model 1*			Model 2†		
	RR	95% CI	*p* value	RR	95% CI	*p* value
Fruit and/or vegetable consumption						
<400 g/day (n = 76)	1.00			1.00		
≥400 g/day (n = 103)	0.41	[0.17–1.03]	0.06	0.49	[0.18–1.32]	0.16
Fruit consumption						
<240 g/day (n = 110)	1.00			1.00		
≥240 g/day (n = 69)	0.57	[0.21–1.58]	0.28	0.80	[0.28–2.31]	0.68
Vegetable consumption						
<240 g/day (n = 94)	1.00			1.00		
≥240 g/day (n = 85)	0.37	[0.13–1.02]	0.06	**0.35**	**[0.13–0.98]**	**0.04**

Level of significance: p = 0.05 (unadjusted [Model 1] and adjusted Poisson regression models [Model 2]). Results for which the 95% CI excludes the null are shown in bold font.
HCC, hepatocellular carcinoma; RR, relative risk.
*Unadjusted Poisson regression models.
†Poisson regression models adjusted on dietary energy, age, sex, cirrhosis causes, history of diabetes, smoking status, alcohol consumption, body mass index, level of physical activity, coffee consumption, education level, occupational status, country or region of birth using inverse probability of treatment weighting with propensity scores.

Table 4. Associations between fruit and/or vegetable consumption and incident HCC or death related to a liver disease (n = 179).

	Model 1*			Model 2†		
	RR	95% CI	*p* value	RR	95% CI	*p* value
Fruit and/or vegetable consumption						
<400 g/day (n = 76)	1.00			1.00		
≥400 g/day (n = 103)	**0.47**	**[0.24–0.95]**	**0.03**	0.63	[0.31–1.30]	0.21
Fruit consumption						
<240 g/day (n = 110)	1.00			1.00		
≥240 g/day (n = 69)	0.94	[0.46–1.90]	0.86	1.40	[0.67–2.94]	0.37
Vegetable consumption						
<240 g/day (n = 94)	1.00			1.00		
≥240 g/day (n = 85)	0.53	[0.26–1.069]	0.09	0.63	[0.31–1.26]	0.19

Level of significance: p = 0.05 (unadjusted [Model 1] and adjusted Poisson regression models [Model 2]). Results for which the 95% CI excludes the null are shown in bold font. HCC, hepatocellular carcinoma; RR, relative risk.

*Unadjusted Poisson regression models.

†Poisson regression models adjusted on dietary energy, age, sex, cirrhosis causes, history of diabetes, smoking status, alcohol consumption, body mass index, level of physical activity, coffee consumption, education level, occupational status, country or region of birth using inverse probability of treatment weighting with propensity scores.

results of our study. This difference in effect size could be explained by the fact that the European study included healthy men and women, whereas our study included patients with cirrhosis. It suggests a potential higher benefit for patients with cirrhosis to consume sufficient vegetables to reduce the risk of HCC, compared with the general population. It could also explain why the results of a case-control Greek study including patients without liver disease contrasted with our findings.[9] The absence of evidence of an association between fruit consumption and the risk of HCC (and death related to liver disease) is consistent with the results of a systematic review and could suggest different mechanisms in the effect of fruit consumption and HCC risk, and vegetable consumption and HCC risk.[7] Note that the slight discrepancy between the Poisson and Cox models could be explained by the inherently different methods of estimating the association parameters between the models (*i.e.* RR for the Poisson model and HR for the Cox model). The fact that the effect sizes were close between the two models gives confidence in the reliability of our results.

Beginning as far back as 1997, the World Cancer Research Fund and the American Institute for Cancer Research began assessing the relationship between fiber consumption and cancer risk. Their latest report, published in 2018, concludes that consuming fiber-rich foods is linked to a reduced risk of colorectal cancer, supported by a probable level of evidence.[3] In 2015, a collective expertise report coordinated by the French National Cancer Institute (INCa) described the level of evidence as convincing for colorectal cancer and probable for breast cancer with mechanisms that could be common to HCC.[39] A diet rich in fiber may influence various mechanisms involved in cancer development, including reduced insulin secretion and insulin resistance, lower blood levels of hormones and growth factors associated with cell proliferation, and alterations in the intestinal microbiota that produce short-chain fatty acids with anti-inflammatory and anti-proliferative properties. Additionally, consuming fiber-rich foods can indirectly reduce the risk of overweight and obesity, which are predictive factors for HCC development in cirrhosis. However, this last argument may not be valid in the context of our study, as the risk of HCC associated with vegetable consumption has been adjusted for body mass index. Furthermore, the antioxidants present in vegetables may provide protective benefits against the occurrence of HCC.[40] Oxidative stress is a key mechanism in hepatocarcinogenesis, where reactive oxygen species (ROS) contribute to cellular damage and the progression of liver carcinogenesis by forming lipid peroxides.[41] ROS accumulation leads to structural and functional DNA alterations that can trigger cell cycle arrest or apoptosis, severely impacting gene functions such as replication and transcription, thus playing a crucial role in cancer initiation and promotion.[42] Additionally, ROS accumulation induces the production of various cytokines and upregulates angiogenesis and the metastatic process.[43] Lastly, micronutrients found in vegetables could have anti-inflammatory effects associated with a reduction of HCC risk.[44]

This study had strengths and limitations that should be considered when interpreting the results. The longitudinal design of the study, the strength (*i.e.* effect size) of associations, and the inclusion of patients with only cirrhosis are strengths that could give confidence in the validity of the results and contribute to the understanding of the effects of fruit and/or vegetable consumption on incident HCC and related liver disease among patients with cirrhosis. This study sample may be too small and may have prevented us from highlighting a significant association between fruit and/or vegetable consumption and HCC, and significant associations using the Cox model. In fact, based on the RR for fruit and/or vegetable consumption (RR = 0.49), the proportion of incident HCC (11.2%), the proportion of patients with fruit and/or vegetable consumption ≥400 g/day (57.5%), and assuming 80% power and 5% alpha risk, a total of 363 patients would need to be included to detect a significant association.[45] Adjustment in a small sample size with a low number of events increases the likelihood of overfitting. Despite the use of propensity scores to adjust for multiple variables, we cannot be certain that all potential confounders have been accounted for in this study and there may be residual confounding. The requirement for histological evidence of cirrhosis for the inclusion of patients in our national prospective cohorts limited the recruitment but this criterion allowed for the selection of a homogeneous population with an unequivocal diagnosis of cirrhosis. Our results may not be generalizable to other regions of France, given the precarious nature of the patient population in the department where the study was conducted (low proportion of working and highly educated patients, high proportion of migrants). The measures of fruit and/or vegetable consumption, and characteristics of patients during a face-to-face interview could be subject to social desirability bias and bias related to the subjectivity of the dietician. However, the interviews were

conducted by a dietician trained for the research, which could have limited the bias. The use of a food and frequency questionnaire could have led to an overestimation of fruit and/or vegetable consumption. This could explain the relatively high proportion of patients meeting the French and WHO thresholds (*i.e.* 57.5%) compared with the general French population (*i.e.* 41.7%). In addition, the overestimation is likely because the patients came from the northeastern suburbs of Paris, a region characterized by low socioeconomic levels and a high proportion of immigrants. Patients' socioeconomic status was measured by education level and occupational status. It would have been useful to supplement these variables with a measure of economic status, such as income, to fully capture the socioeconomic status of patients. As income was not collected in the study, this could result in potential residual confounding.

Conclusions

Other than an insufficient fruit and/or vegetable consumption in 42.5% of our patients with cirrhosis, a 65% reduction of HCC occurrence was observed in those with vegetable consumption ≥240 g/day. External validation is required to confirm these results, and larger studies are needed to assess the benefits of fruit regarding the risk of HCC, and to provide evidence for promoting fruit and vegetable consumption in patients with cirrhosis.

Affiliations

[1]*Université Sorbonne Paris Nord and Université Paris Cité, INSERM, INRAE, CNAM, Center of Research in Epidemiology and StatisticS (CRESS), Nutritional Epidemiology Research Team (EREN), Bobigny, France;* [2]*Liver Unit, Avicenne Hospital, Assistance Publique-Hôpitaux de Paris (AP-HP), Bobigny, France;* [3]*Université Sorbonne Paris Nord, Bobigny, France;* [4]*INSERM UMR S-1138, équipe FunGeST Centre de Recherche des Cordeliers Sorbonne Université, Paris, France;* [5]*Public Health Department, Avicenne Hospital, Assistance Publique-Hôpitaux de Paris (AP-HP), Bobigny, France*

Abbreviations

HCC, hepatocellular carcinoma; HR, hazard ratio; MASLD, metabolic dysfunction-associated steatotic liver disease; ROS, reactive oxygen species; RR, relative risk.

Financial support

The ALICIR study is supported by ANRS (France Recherche Nord & Sud Sida-HIV Hépatites). CIRRAL has been funded by the National Institute of Cancer (INCa, Institut National du Cancer), the ARC Foundation, and ANRS. The ANRS CO12 CirVir cohort is sponsored and funded by ANRS. This analysis was conducted as part of the GENIAL project ('Understanding Gene ENvironment Interaction in ALcohol-related hepatocellular carcinoma'), which is funded by the European Union within the Horizon Europe programme under agreement (No 101096312).

Conflicts of interest

PN has received grant support from ASTRA ZENECA, BMS, and EISAI. He has also received consultancy fees and payments or honoraria for lectures, presentations, speakers bureaus, manuscript preparation, or educational activities from GILEAD, ASTRA ZENECA, BMS, and ROCHE. He has also received support for attendance at meetings and/or travel from ROCHE and ASTRA ZENECA. The other authors have nothing to disclose.

Please refer to the accompanying ICMJE disclosure forms for further details.

Authors' contributions

Conceptualization, NG-C, CJ. Methodology: MF, ZZ, NG-C, CJ. Visualization: MF. Data curation: MF, ZZ. Formal analysis: MF, ZZ. Writing – original draft preparation: MF. Writing – review and editing: ZZ, SD, LKF, AB, PN, MT, NG-C, CJ. Supervision, CJ. Read and approved the final manuscript: all authors.

Data availability statement

Data analyzed during this study are not available because of legal restrictions.

Acknowledgements

This work is dedicated to the memory of Prof. Jean-Claude Trinchet. We thank all the staff who participated in the data collection and data management.

Supplementary data

Supplementary data to this article can be found online at https://doi.org/10.1016/j.jhepr.2025.101355.

References

Author names in bold designate shared co-first authorship

[1] Sung H, Ferlay J, Siegel RL, et al. Global Cancer Statistics 2020: GLOBOCAN estimates of incidence and mortality worldwide for 36 cancers in 185 countries. CA Cancer J Clin 2021;71:209–249.
[2] Villanueva A. Hepatocellular carcinoma. N Engl J Med 2019;380:1450–1462.
[3] World Cancer Research Fund/American Institute for Cancer Research. Diet, nutrition, physical activity and cancer: a global perspective. WCRF/AICR; 2018.
[4] Kennedy OJ, Roderick P, Buchanan R, et al. Coffee, including caffeinated and decaffeinated coffee, and the risk of hepatocellular carcinoma: a systematic review and dose–response meta-analysis. BMJ Open 2017;7:e013739.
[5] Bhurwal A, Ratta P, Yoshitake S, et al. Inverse association of coffee with liver cancer development: an updated systematic review and meta-analysis. J Gastrointestin Liver Dis 2020;29:421–428.
[6] DiJoseph K, Thorp A, Harrington A, et al. Physical activity and risk of hepatocellular carcinoma: a systematic review and meta-analysis. Dig Dis Sci 2023;68:1051–1059.
[7] George ES, Sood S, Broughton A, et al. The association between diet and hepatocellular carcinoma: a systematic review. Nutrients 2021;13:172.
[8] Bamia C, Lagiou P, Jenab M, et al. Fruit and vegetable consumption in relation to hepatocellular carcinoma in a multi-centre, European cohort study. Br J Cancer 2015;112:1273.
[9] Kuper H, Tzonou A, Lagiou P, et al. Diet and hepatocellular carcinoma: a case-control study in Greece. Nutr Cancer 2000;38:6–12.
[10] Yang W, Ma Y, Liu Y, et al. Association of intake of whole grains and dietary fiber with risk of hepatocellular carcinoma in US adults. J Am Med Assoc Oncol 2019;5:879.
[11] Zhao L, Jin L, Petrick JL, et al. Specific botanical groups of fruit and vegetable consumption and liver cancer and chronic liver disease mortality: a prospective cohort study. Am J Clin Nutr 2023;117:278–285.
[12] Kurozawa Y, Ogimoto I, Shibata A, et al. Dietary habits and risk of death due to hepatocellular carcinoma in a large scale cohort study in Japan. Univariate analysis of JACC study data. Kurume Med J 2004;51:141–149.
[13] Buscail C, Bourcier V, Fezeu LK, et al. Eating patterns in patients with compensated cirrhosis: a case-control study. Nutrients 2018;10:60.
[14] Elm E von, Altman DG, Egger M, et al. The Strengthening the Reporting of Observational Studies in Epidemiology (STROBE) statement: guidelines for reporting observational studies. Lancet 2007;370:1453–1457.
[15] Trinchet J-C, Bourcier V, Chaffaut C, et al. Complications and competing risks of death in compensated viral cirrhosis (ANRS CO12 CirVir prospective cohort). Hepatology 2015;62:737–750.
[16] Ganne-Carrié N, Chaffaut C, Bourcier V, et al. Estimate of hepatocellular carcinoma incidence in patients with alcoholic cirrhosis. J Hepatol 2018;69:1274–1283.
[17] Bruix J, Sherman M, Practice Guidelines Committee, American Association for the Study of Liver Diseases. Management of hepatocellular carcinoma. Hepatology 2005;42:1208–1236.
[18] Bruix J, Sherman M, American Association for the Study of Liver Diseases. Management of hepatocellular carcinoma: an update. Hepatology 2011;53:1020–1022.
[19] De Franchis R, Bosch J, Garcia-Tsao G, et al. Baveno VII – renewing consensus in portal hypertension. J Hepatol 2022;76:959–974.
[20] Kesse-Guyot E, Castetbon K, Touvier M, et al. Relative validity and reproducibility of a food frequency questionnaire designed for French adults. Ann Nutr Metab 2010;57:153–162.

[21] Hercberg S, Deheeger M, Preziosi P, et al. SU.VI.MAX. Portions Alimentaires. Editions polytechnica. Quantités. Paris: Manuel Photos pour l'Estimation desQuantités; 2002. 2002.
[22] Nutrition and Food Safety (NFS). Diet, nutrition and the prevention of chronic diseases: report of a joint WHO/FAO expert consultation. Geneva: World Health Organization; 2002.
[23] Ministère des solidarités et de la santé. Programme national nutrition-santé 2019-2023. France: Ministère des Solidarités et de la Santé. 2019.
[24] CDC. Defining adult overweight and obesity. Centers for Disease Control and Prevention; 2022. https://www.cdc.gov/obesity/basics/adult-defining.html. [Accessed 7 December 2023].
[25] Craig C, Marshall A, Sjöström M, et al. International physical activity questionnaire: 12-country reliability and validity. Med Sci Sports Exerc 2003;35:1381–1395.
[26] Scoring protocol for the International Physical Activity Questionnaire (IPAQ) n.d. https://sites.google.com/view/ipaq/score (accessed January 2, 2024).
[27] Rinella ME, Lazarus JV, Ratziu V, et al. A multisociety Delphi consensus statement on new fatty liver disease nomenclature. J Hepatol 2023;79:1542–1556.
[28] Vittinghoff E, McCulloch CE. Relaxing the rule of ten events per variable in logistic and cox regression. Am J Epidemiol 2007;165:710–718.
[29] Austin PC. An introduction to propensity score methods for reducing the effects of confounding in observational studies. Multivariate Behav Res 2011;46:399–424.
[30] Kuss O, Blettner M, Börgermann J. Propensity Score: an alternative method of analyzing treatment effects. Dtsch Arztebl Int 2016;113:597–603.
[31] Stuart EA. Matching methods for causal inference: a review and a look forward. Stat Sci 2010;25:1–21.
[32] European Association for the Study of the Liver. EASL Clinical Practice Guidelines on nutrition in chronic liver disease. J Hepatol 2019;70:172.
[33] Pashayee-khamene F, Saber-firoozi M, Hatami B, et al. Food groups intake of cirrhotic patients, comparison with the nutritional status and disease stage. Gastroenterol Hepatol Bed Bench 2019;12:226.
[34] Khan R, Ahmed A, Ismail FW, et al. Perception and knowledge about dietary intake in patients with cirrhosis and its relationship with the level of education. J Coll Physicians Surg Pak 2012;22:435–439.
[35] Volk ML, Fisher N, Fontana RJ. Patient knowledge about disease self-management in cirrhosis. Am J Gastroenterol 2013;108:302–305.
[36] Valery PC, Bernardes CM, Mckillen B, et al. The patient's perspective in cirrhosis: unmet supportive care needs differ by disease severity, etiology, and age. Hepatol Commun 2021;5:891. https://doi.org/10.1002/hep4.1681.
[37] Chang Y, Lee S, Lee M, et al. Nutritional status of Korean male patients with alcoholic and viral cirrhosis. Asia Pac J Clin Nutr 2003;12:203–208.
[38] Alavinejad P, Hajiani E, Danyaee B, et al. The effect of nutritional education and continuous monitoring on clinical symptoms, knowledge, and quality of life in patients with cirrhosis. Gastroenterol Hepatol Bed Bench 2019;12:17–24.
[39] INCa. Nutrition et prévention primaire des cancers : actualisation des données. France; 2015.
[40] World Cancer Research Fund. Wholegrains, vegetables and fruit and the risk of cancer. WCRF; 2018.
[41] Kaulmann A, Bohn T. Carotenoids, inflammation, and oxidative stress–implications of cellular signaling pathways and relation to chronic disease prevention. Nutr Res 2014;34:907–929.
[42] Khansari N, Shakiba Y, Mahmoudi M. Chronic inflammation and oxidative stress as a major cause of age- related diseases and cancer. Recent Pat Inflamm Allergy Drug Discov 2006;3:73–80.
[43] Reuter S, Gupta SC, Chaturvedi MM, et al. Oxidative stress, inflammation, and cancer: how are they linked? Free Rad Biol Med 2010;49:1603–1616.
[44] Yang W, Sui J, Ma Y, et al. High dietary intake of vegetable or poly-unsaturated fats is associated with reduced risk of hepatocellular carcinoma. Clin Gastroenterol Hepatol 2020;18:2775. 83.e11.
[45] Serdar CC, Cihan M, Yücel D, et al. Sample size, power and effect size revisited: simplified and practical approaches in pre-clinical, clinical and laboratory studies. Biochem Med (Zagreb) 2020;31:010502.

Keywords: Eating behavior; Liver cancer; Cohort study.
Received 27 May 2024; received in revised form 4 February 2025; accepted 6 February 2025; Available online 13 February 2025

Journal of Hepatology, Volume ■

Supplemental information

Associations between fruit and vegetable consumption and HCC occurrence in patients with cirrhosis

Florian Manneville, Zineb Zouakia, Séverine Donneger, Leopold K. Fezeu, Alice Bellicha, Pierre Nahon, Mathilde Touvier, Nathalie Ganne-Carrié, and Chantal Julia

Associations between fruit and vegetable consumption and incident hepatocellular carcinoma among patients with cirrhosis

Florian Manneville, Zineb Zouakia, Séverine Donneger, Leopold K. Fezeu, Alice Bellocchi, Pierre Nahon, Mathilde Touvier, Nathalie Ganne-Carrié, Chantal Julia

Table of contents

Fig. S1. 2

Fig. S2. 4

Fig. S3. 6

Table S1. 8

Table S2. 9

Table S3. 10

Table S4 11

Table S5. 13

Table S6. 14

Table S7. 15

Table S8 16

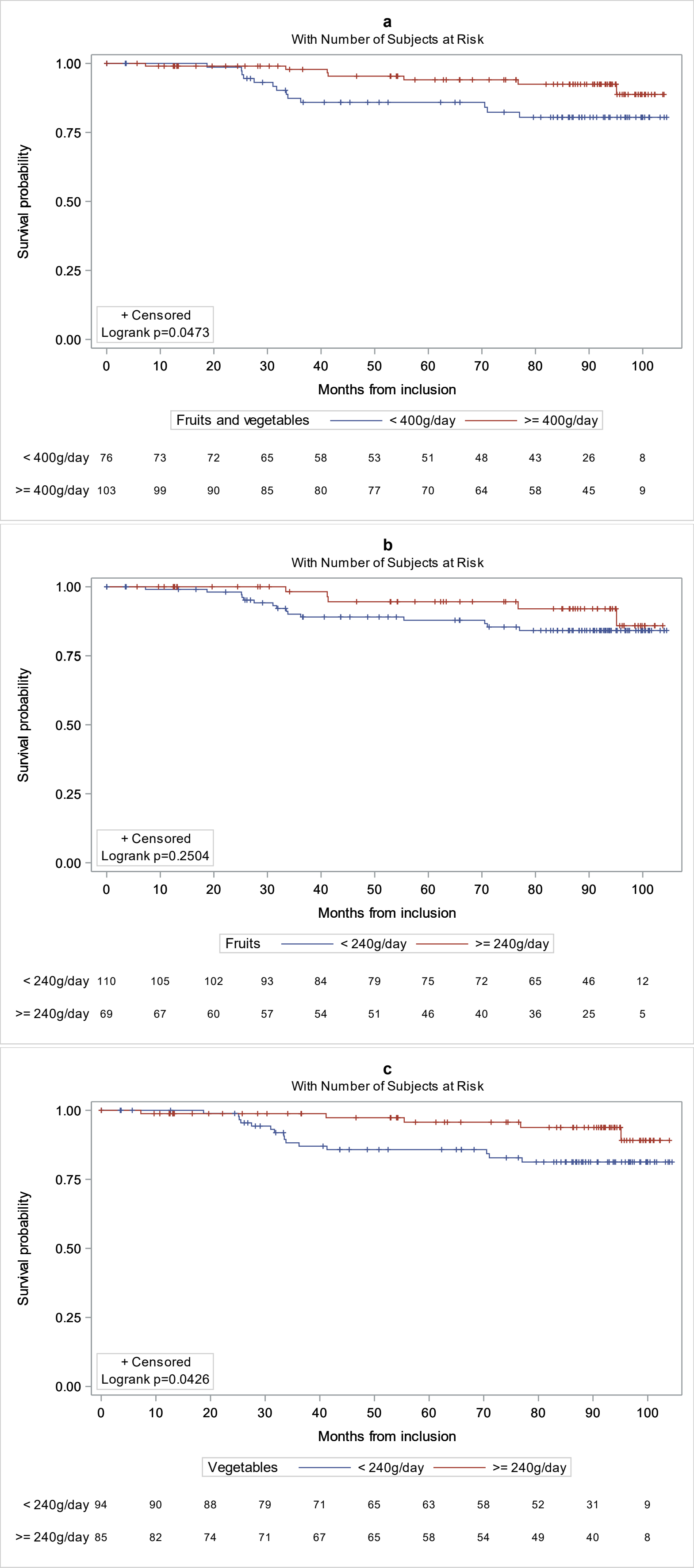
a
With Number of Subjects at Risk
Survival probability
+ Censored
Logrank p=0.0473
Months from inclusion
Fruits and vegetables
< 400g/day
>= 400g/day
< 400g/day 76 73 72 65 58 53 51 48 43 26 8
>= 400g/day 103 99 90 85 80 77 70 64 58 45 9
b
With Number of Subjects at Risk
Survival probability
+ Censored
Logrank p=0.2504
Months from inclusion
Fruits
< 240g/day
>= 240g/day
< 240g/day 110 105 102 93 84 79 75 72 65 46 12
>= 240g/day 69 67 60 57 54 51 46 40 36 25 5
c
With Number of Subjects at Risk
Survival probability
+ Censored
Logrank p=0.0426
Months from inclusion
Vegetables
< 240g/day
>= 240g/day
< 240g/day 94 90 88 79 71 65 63 58 52 31 9
>= 240g/day 85 82 74 71 67 65 58 54 49 40 8

Fig. S1. Kaplan-Meier curves for incident HCC among patients with liver cirrhosis who consumed a) fruit and/or vegetable, b) fruit, and c) vegetable at or above thresholds (n=179). Level of significance: p= 0.05 (logrank test).

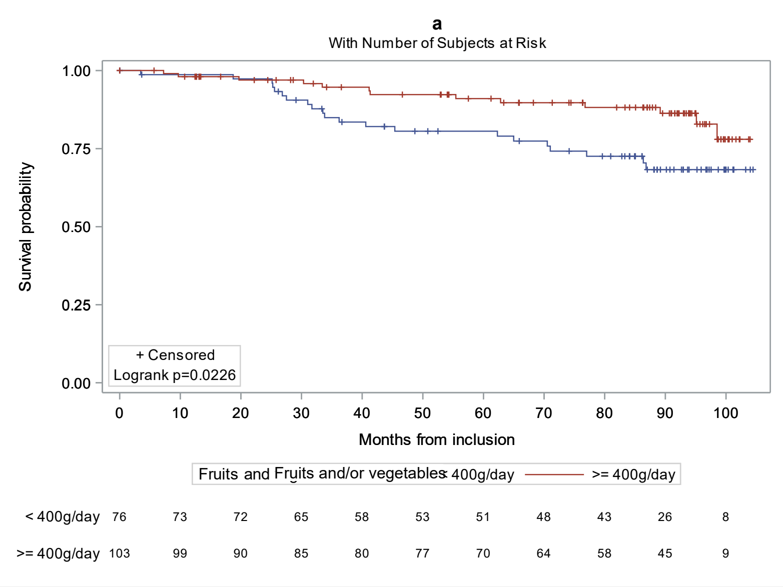
a
With Number of Subjects at Risk
Survival probability
1.00
0.75
0.50
0.25
0.00
+ Censored
Logrank p=0.0226
0 10 20 30 40 50 60 70 80 90 100
Months from inclusion
Fruits and Fruits and/or vegetables < 400g/day >= 400g/day
< 400g/day 76 73 72 65 58 53 51 48 43 26 8
>= 400g/day 103 99 90 85 80 77 70 64 58 45 9

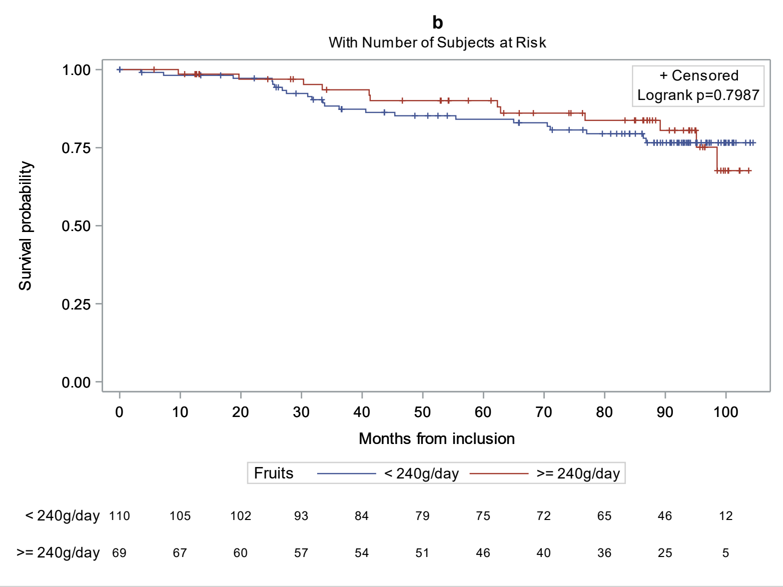
b
With Number of Subjects at Risk
Survival probability
1.00
0.75
0.50
0.25
0.00
+ Censored
Logrank p=0.7987
0 10 20 30 40 50 60 70 80 90 100
Months from inclusion
Fruits < 240g/day >= 240g/day
< 240g/day 110 105 102 93 84 79 75 72 65 46 12
>= 240g/day 69 67 60 57 54 51 46 40 36 25 5

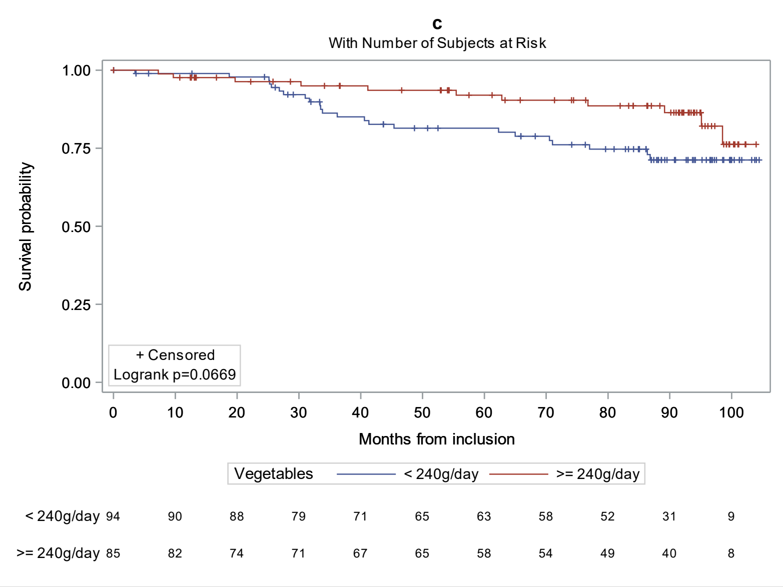
c
With Number of Subjects at Risk
Survival probability
1.00
0.75
0.50
0.25
0.00
+ Censored
Logrank p=0.0669
0 10 20 30 40 50 60 70 80 90 100
Months from inclusion
Vegetables < 240g/day >= 240g/day
< 240g/day 94 90 88 79 71 65 63 58 52 31 9
>= 240g/day 85 82 74 71 67 65 58 54 49 40 8

Fig. S2. Kaplan-Meier curves for incident HCC or death related to a liver disease among patients with liver cirrhosis who consumed a) fruit and/or vegetable, b) fruit, and c) vegetable at or above thresholds (n=179). Level of significance: p= 0.05 (logrank test).

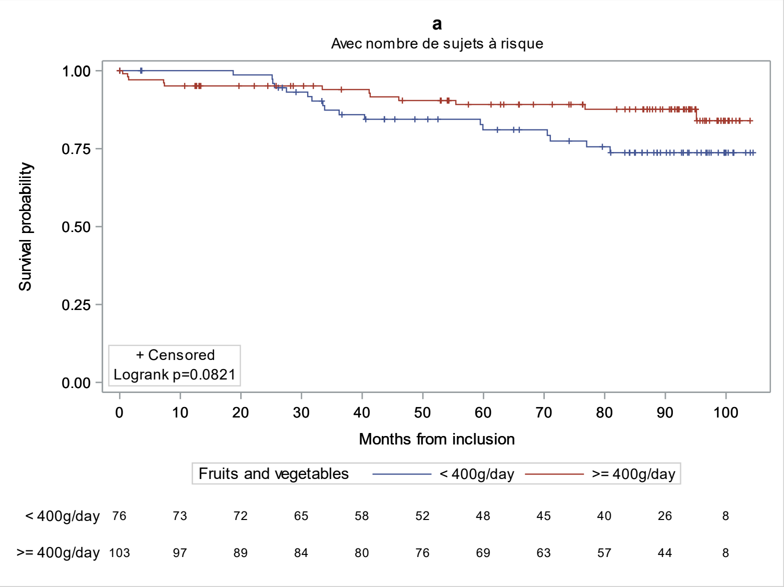
a
Avec nombre de sujets à risque
Survival probability
1.00
0.75
0.50
0.25
0.00
+ Censored
Logrank p=0.0821
0
10
20
30
40
50
60
70
80
90
100
Months from inclusion
Fruits and vegetables
< 400g/day
>= 400g/day
< 400g/day 76 73 72 65 58 52 48 45 40 26 8
>= 400g/day 103 97 89 84 80 76 69 63 57 44 8

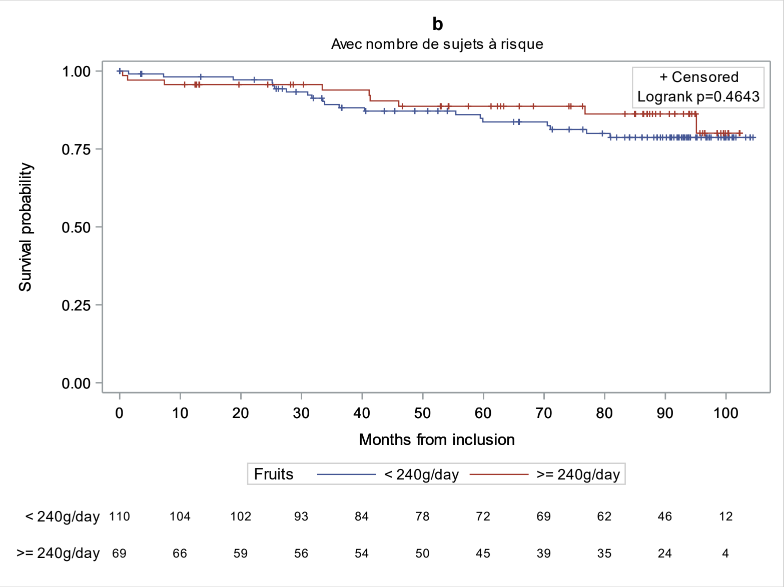
b
Avec nombre de sujets à risque
Survival probability
1.00
0.75
0.50
0.25
0.00
+ Censored
Logrank p=0.4643
0
10
20
30
40
50
60
70
80
90
100
Months from inclusion
Fruits
< 240g/day
>= 240g/day
< 240g/day 110 104 102 93 84 78 72 69 62 46 12
>= 240g/day 69 66 59 56 54 50 45 39 35 24 4

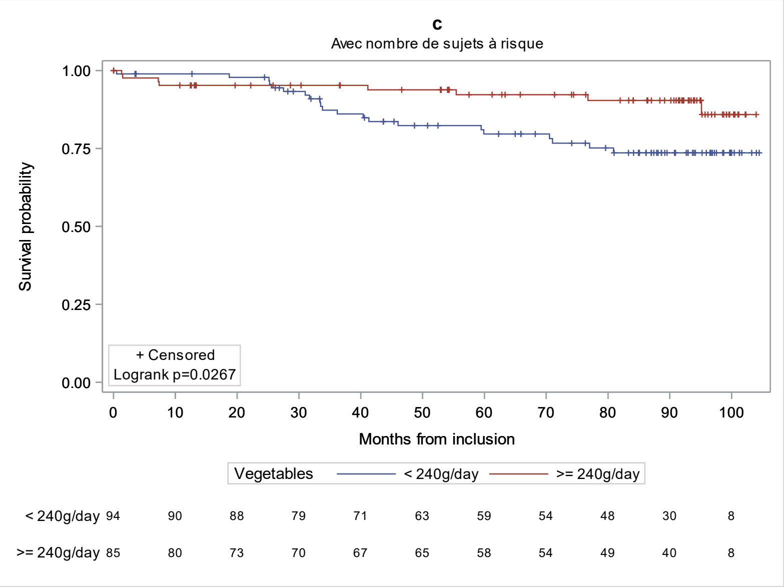
c
Avec nombre de sujets à risque
Survival probability
1.00
0.75
0.50
0.25
0.00
+ Censored
Logrank p=0.0267
0
10
20
30
40
50
60
70
80
90
100
Months from inclusion
Vegetables
< 240g/day
>= 240g/day
< 240g/day 94 90 88 79 71 63 59 54 48 30 8
>= 240g/day 85 80 73 70 67 65 58 54 49 40 8

Fig. S3. Kaplan-Meier curves for incident all hepatic events (HCC and decompensation defined according to BAVENO VII) among patients with liver cirrhosis who consumed a) fruit and/or vegetable, b) fruit, and c) vegetable at or above thresholds (n=179). Level of significance: p= 0.05 (logrank test).

Table S1. Comparisons of patients' baseline characteristics according to incident HCC (n= 179). Level of significance: p= 0.05 (Chi-squared test or Fisher's exact test for categorical variables and Wilcoxon rank-sum test the quantitative variable).

		%		
		HCC+ (n= 20)	HCC- (n= 159)	p-value
Sex	Male	80.0	69.8	0.34
	Female	20.0	30.2	
Age (years)	<50	5.0	17.0	0.05
	[50-60[	35.0	35.2	
	[60-70[	55.0	28.9	
	>= 70	5.0	18.9	
Body mass index (kg/m²)	<25	40.0	37.1	0.92
	[25-30[	40.0	39.0	
	≥30	20.0	23.9	
Education level	No high school diploma	35.0	39.6	0.73
	High school diploma	50.0	40.9	
	University degree	15.0	19.5	
Marital status	Single	25.0	33.3	0.45
	Cohabiting	75.0	66.7	
Occupational status	Employed	25.0	39.6	0.35
	Not in the labor force	60.0	52.2	
	Sick leave	15.0	8.2	
Smoking status	Former smoker or non-smoker	55.0	74.8	0.06
	Smoker	45.0	25.2	
Alcohol consumption	Never	65.0	59.1	0.50
	Occasionally	15.0	26.4	
	Regularly	20.0	14.5	
Level of total physical activity	High	15.0	13.8	0.15
	Moderate	30.0	52.8	
	Low	45.0	23.9	
	Missing	10.0	9.4	
Liver cirrhosis causes	Alcoholic	60.0	42.1	0.13
	Viral	40.0	57.9	
Country or region of birth	Africa	10.0	16.5	0.09
	Asia	10.0	10.8	
	Europe	5.0	14.6	
	France	75.0	44.0	
	Maghreb	0.0	14.6	
Coffee consumption (g/day)	0	15.0	23.3	0.68
	]1-93[	25.0	25.2	
	≥93	60.0	51.6	
Dietary energy (kcal)	Median (Q1-Q3)	1890.3 (1499.6-2487.4)	1933.6 (1286.5-2626.1)	0.83
History of diabetes	No	70.0	71.1	0.92
	Yes	30.0	28.9	

p-values were obtained using Chi-squared test or Fisher's exact test for categorical variables and Wilcoxon rank-sum test for the quantitative variable.

Table S2. Comparisons of patients' baseline characteristics according to fruit consumption (n= 179). Level of significance: p= 0.05 (Chi-squared test or Fisher's exact test for categorical variables and Wilcoxon rank-sum test the quantitative variable).

		%		
		**<240 g/day (n= 110)**	**>= 240 g/day (n= 69)**	**p-value**
Sex	Male	69.1	73.9	0.49
	Female	30.9	26.1	
Age (years)	<50	14.5	17.3	0.65
	[50-60[	38.2	30.4	
	[60-70[	28.2	37.7	
	>= 70	19.0	14.4	
Body mass index (kg/m²)	<25	40.0	33.3	0.44
	[25-30[	35.4	44.9	
	≥30	24.5	21.7	
Education level	No high school diploma	38.2	40.6	0.83
	High school diploma	43.6	39.1	
	University degree	18.2	20.3	
Marital status	Single	32.7	31.9	0.91
	Cohabiting	67.3	68.1	
Occupational status	Employed	35.4	42.0	0.42
	Not in the labor force	53.6	52.2	
	Sick leave	10.9	5.8	
Smoking status	Former smoker or non-smoker	66.4	82.6	0.02
	Smoker	33.6	17.4	
Alcohol consumption	Never	61.8	56.5	0.77
	Occasionally	23.6	27.5	
	Regularly	14.5	15.9	
Level of total physical activity	High	10.0	20.3	0.24
	Moderate	50.9	49.3	
	Low	29.1	21.7	
	Missing	10.0	8.7	
Liver cirrhosis causes	Alcoholic	42.7	46.4	0.63
	Viral	57.3	53.6	
Country or region of birth	Africa	15.4	15.9	0.01
	Asia	10.0	11.6	
	Europe	8.2	21.7	
	France	56.4	33.3	
	Maghreb	10.0	17.4	
Coffee consumption (g/day)	0	22.7	21.7	0.84
	]1-90[	23.6	27.5	
	≥90	53.6	50.7	
Dietary energy (kcal)	Median (Q1-Q3)	1768.5 (1339.4-2360.6)	2239.3 (1744.2-2772.8)	0.002
History of diabetes	No	71.8	69.6	0.75
	Yes	28.2	30.4	

p-values were obtained using Chi-squared test or Fisher's exact test for categorical variables and Wilcoxon rank-sum test the quantitative variable.

Table S3. Comparisons of patients' baseline characteristics according to vegetable consumption (n= 179). Level of significance: p= 0.05 (Chi-squared test or Fisher's exact test for categorical variables and Wilcoxon rank-sum test the quantitative variable).

		%		
		**<240 g/day (n= 94)**	**>= 240 g/day (n= 85)**	**p-value**
Sex	Male	70.2	71.8	0.82
	Female	29.8	28.2	
Age (years)	<50	12.7	18.8	0.33
	[50-60[	40.4	29.4	
	[60-70[	27.7	36.5	
	>= 70	19.2	15.3	
Body mass index (kg/m²)	<25	36.2	38.8	0.79
	[25-30[	38.3	40.0	
	≥30	25.5	21.2	
Education level	No high school diploma	38.3	40.0	0.88
	High school diploma	43.6	40.0	
	University degree	18.1	20.0	
Marital status	Single	31.9	32.9	0.88
	Cohabiting	68.1	67.1	
Occupational status	Employed	37.2	38.6	0.94
	Not in the labor force	53.2	52.9	
	Sick leave	9.6	8.2	
Smoking status	Former smoker or non-smoker	68.1	77.6	0.15
	Smoker	31.9	22.3	
Alcohol consumption	Never	57.4	62.3	0.71
	Occasionally	25.5	24.7	
	Regularly	17.0	12.9	
Level of total physical activity	High	8.5	20.0	0.08
	Moderate	50.0	50.6	
	Low	31.9	20.0	
	Missing	9.6	9.4	
Liver cirrhosis causes	Alcoholic	46.8	41.2	0.45
	Viral	53.2	58.8	
Country or region of birth	Africa	16.0	15.3	0.40
	Asia	7.4	14.1	
	Europe	12.8	14.1	
	France	53.2	41.2	
	Maghreb	10.6	15.3	
Coffee consumption (g/day)	0	26.6	17.6	0.22
	]1-90[	26.6	23.5	
	≥90	46.8	58.8	
Dietary energy (kcal)	Median (Q1-Q3)	1890.3 (1499.6-2487.4)	2138.1 (1709.4-2772.8)	0.001
History of diabetes	No	72.3	69.4%	0.67
	Yes	27.7	30.6%	

p-values were obtained using Chi-squared test or Fisher's exact test for categorical variables and Wilcoxon rank-sum test for the quantitative variable.

Table S4. Standardized differences in confounders between patients below the fruit and/or vegetable consumption threshold and patients above or at the fruit and/or vegetable consumption threshold before and after the use of the propensity score

Cofounder	Fruit and/or vegetable consumption		Fruit consumption		Vegetable consumption	
	Standardized difference before propensity score	Standardized difference after propensity score	Standardized difference before propensity score	Standardized difference after propensity score	Standardized difference before propensity score	Standardized difference after propensity score
Dietary energy	0.37732	0.07157	0.38451	0.08698	0.46601	0.03458
Sex	0.14682	-0.00425	0.10698	0.01798	0.03420	0.04769
Education level	0.14634	0.06625	0.10215	0.18309	0.08338	0.04200
Occupational status	0.14869	0.08089	0.20823	0.27564	0.07419	0.03703
Smoking status	-0.37024	-0.11248	-0.37929	0.09521	-0.21630	-0.15997
Level of physical activity	0.41905	0.05837	0.29950	0.12910	0.37952	0.09919
Liver cirrhosis causes	0.24488	0.05230	0.29004	-0.07570	0.33316	-0.00798
Country or region of birth	0.58106	0.12689	0.54822	0.17632	0.31564	0.12591
Alcohol consumption	0.08711	0.03366	0.10524	0.08585	0.12377	0.03301
Body mass index	0.12274	0.02317	0.20665	0.06267	0.11880	0.06549
Age	0.50918	0.22750	0.29332	0.11682	0.35780	0.07760
History of diabetes	-0.12511	-0.04817	-0.02910	-0.03267	-0.07467	-0.03966

Cofounder	Fruit and/or vegetable consumption		Fruit consumption		Vegetable consumption	
	Standardized difference before propensity score	Standardized difference after propensity score	Standardized difference before propensity score	Standardized difference after propensity score	Standardized difference before propensity score	Standardized difference after propensity score
Coffee consumption	0.17707	0.04795	0.09145	0.12184	0.26148	0.04922

Table S5. Associations between fruit and/or vegetable consumption and incident all hepatic events (HCC and decompensation defined according to BAVENO VII) (n= 179). Level of significance: p= 0.05 (unadjusted [Model 1] and adjusted Poisson regression models [Model 2]).

	Model 1[a]			Model 2[b]		
	RR	95% CI	p	RR	95% CI	p
Fruit and/or vegetable consumption						
<400 g/day (n= 76)	1.00			1.00		
≥400 g/day (n=103)	0.52	[0.25- 1.08]	0.08	0.72	[0.34- 1.54]	0.39
Fruit consumption						
<240 g/day (n= 110)	1.00			1.00		
≥240 g/day (n= 69)	0.75	[0.34- 1.64]	0.47	1.03	[0.46- 2.34]	0.94
Vegetable consumption						
<240 g/day (n= 94)	1.00			1.00		
≥240 g/day (n= 85)	**0.40**	**[0.18- 0.91]**	**0.03**	0.49	[0.22- 1.08]	0.08

Abbreviations: RR, relative risk; 95% CI, 95% confidence interval.

[a] *Unadjusted Poisson regression models.*

[b] *Poisson regression models adjusted on dietary energy, age, sex, liver cirrhosis causes, history of diabetes, smoking status, alcohol consumption, body mass index, level of physical activity, coffee consumption, education level, occupational status, country or region of birth using inverse probability of treatment weighting with propensity scores.*

Results for which the 95% CI excludes the null are bolded

Table S6. Associations between fruit and/or vegetable consumption and incident HCC: unadjusted and adjusted Cox regression models (n= 179). Level of significance: p= 0.05 (unadjusted [Model 1] and adjusted Cox regression models [Model 2]).

	Model 1[a]			Model 2[b]		
	HR	95% CI	p	HR	95% CI	p
Fruit and/or vegetable consumption						
<400 g/day (n= 76)	1.00			1.00		
≥400 g/day (n=103)	0.41	[0.16- 1.02]	0.055	0.50	[0.18- 1.36]	0.17
Fruit consumption						
<240 g/day (n= 110)	1.00			1.00		
≥240 g/day (n= 69)	0.56	[0.20- 1.53]	0.26	0.87	[0.30- 2.53]	0.80
Vegetable consumption						
<240 g/day (n= 94)	1.00			1.00		
≥240 g/day (n= 85)	0.37	[0.13- 1.01]	0.052	0.36	[0.13- 1.00]	0.051

Abbreviations: HR, hazard ratio; 95% CI, 95% confidence interval.

[a] Unadjusted Cox regression models.

[b] Cox regression models adjusted on dietary energy, age, sex, liver cirrhosis causes, history of diabetes, smoking status, alcohol consumption, body mass index, level of physical activity, coffee consumption, education level, occupational status, country or region of birth using inverse probability of treatment weighting with propensity scores.

Results for which the 95% CI excludes the null are bolded.

Table S7. Associations between fruit and/or vegetable consumption and incident HCC or death related to a liver disease (n= 179). Level of significance: p= 0.05 (unadjusted [Model 1] and adjusted Cox regression models [Model 2]).

	Model 1[a]			Model 2[b]		
	HR	95% CI	p	HR	95% CI	p
Fruit and/or vegetable consumption						
<400 g/day (n= 76)	1.00			1.00		
≥400 g/day (n=103)	**0.46**	**[0.23- 0.91]**	**0.03**	0.63	[0.31- 1.30]	0.21
Fruit consumption						
<240 g/day (n= 110)	1.00			1.00		
≥240 g/day (n= 69)	0.91	[0.45- 1.84]	0.80	1.55	[0.74- 3.25]	0.25
Vegetable consumption						
<240 g/day (n= 94)	1.00			1.00		
≥240 g/day (n= 85)	0.52	[0.25- 1.06]	0.07	0.63	[0.31- 1.26]	0.19

Abbreviations: HR, hazard ratio; 95% CI, 95% confidence interval.

[a] Unadjusted Cox regression models.

[b] Cox regression models adjusted on dietary energy, age, sex, liver cirrhosis causes, history of diabetes, smoking status, alcohol consumption, body mass index, level of physical activity, coffee consumption, education level, occupational status, country or region of birth using inverse probability of treatment weighting with propensity scores.

Results for which the 95% CI excludes the null are bolded

Table S8. Associations between fruit and/or vegetable consumption and incident all hepatic events (HCC and decompensation defined according to BAVENO VII) (n= 179). Level of significance: p= 0.05 (unadjusted [Model 1] and adjusted Cox regression models [Model 2]).

	Model 1[a]			Model 2[b]		
	HR	95% CI	p	HR	95% CI	p
Fruit and/or vegetable consumption						
<400 g/day (n= 76)	1.00			1.00		
≥400 g/day (n=103)	0.52	[0.25- 1.10]	0.09	0.72	[0.34- 1.54]	0.40
Fruit consumption						
<240 g/day (n= 110)	1.00			1.00		
≥240 g/day (n= 69)	0.75	[0.34- 1.64]	0.46	1.06	[0.46- 2.41]	0.89
Vegetable consumption						
<240 g/day (n= 94)	1.00			1.00		
≥240 g/day (n= 85)	**0.41**	**[0.18- 0.93]**	**0.03**	0.50	[0.23- 1.09]	0.08

Abbreviations: HR, hazard ratio; 95% CI, 95% confidence interval.

[a] *Unadjusted Cox regression models.*

[b] *Cox regression models adjusted on dietary energy, age, sex, liver cirrhosis causes, history of diabetes, smoking status, alcohol consumption, body mass index, level of physical activity, coffee consumption, education level, occupational status, country or region of birth using inverse probability of treatment weighting with propensity scores.*

Results for which the 95% CI excludes the null are bolded.
